# Asymmetric connections with starburst amacrine cells underlie the upward motion selectivity of J-type retinal ganglion cells

**DOI:** 10.1371/journal.pbio.3002301

**Published:** 2023-09-18

**Authors:** Bo Wang, Yifeng Zhang

**Affiliations:** Institute of Neuroscience, Center for Excellence in Brain Science and Intelligence Technology, Chinese Academy of Sciences, Shanghai, China; Yale University, UNITED STATES

## Abstract

Motion is an important aspect of visual information. The directions of visual motion are encoded in the retina by direction-selective ganglion cells (DSGCs). ON-OFF DSGCs and ON DSGCs co-stratify with starburst amacrine cells (SACs) in the inner plexiform layer and depend on SACs for their direction selectivity. J-type retinal ganglion cells (J-RGCs), a type of OFF DSGCs in the mouse retina, on the other hand, do not co-stratify with SACs, and how direction selectivity in J-RGCs emerges has not been understood. Here, we report that both the excitatory and inhibitory synaptic inputs to J-RGCs are direction-selective (DS), with the inhibitory inputs playing a more important role for direction selectivity. The DS inhibitory inputs come from SACs, and the functional connections between J-RGCs and SACs are spatially asymmetric. Thus, J-RGCs and SACs form functionally important synaptic contacts even though their dendritic arbors show little overlap. These findings underscore the need to look beyond the neurons’ stratification patterns in retinal circuit studies. Our results also highlight the critical role of SACs for retinal direction selectivity.

## Abstract 2

The directions of visual motion are encoded in the retina by direction-selective ganglion cells. This study shows that the direction-selective J-RGCs in the mouse retina receive spatially asymmetric inputs from starburst amacrine cells even though their dendritic arbors show little overlap.

## Introduction

Most of the synaptic connections in the vertebrate retina are located in the inner plexiform layer (IPL). The IPL is organized into functionally distinct sub-laminas and the neural processes of retinal ganglion cells (RGCs), amacrine cells and bipolar cells often stratify in restricted sub-laminas [1]. It is believed that the restricted stratification helps to bring processes of retinal neurons that need to make connections into close proximity of each other, such as direction-selective ganglion cells (DSGCs) and starburst amacrine cells (SACs). And retinal neurons that do not stratify in the same sub-lamina are considered to have little chance of making direct synaptic connections.

Motion detection is a fundamental task of the visual system. This process starts in the retina [2,3]. DSGCs in the retina are strongly activated by the motion in their preferred direction (PD) but show weak or no response to the motion in the opposite null direction (ND). The mechanisms underlying their direction selectivity are understood to a large extent, and SAC is the most critical component [4,5]. SACs form direct synaptic connections to ON-OFF and ON DSGCs [6] and are direction-selective (DS) themselves, preferring centrifugal to centripetal motion [7]. They have compartmentalized dendrites, different sectors of which prefer different motion directions and output to different DSGCs [7,8]. The connections between DSGCs and SACs are spatially selective: a DSGC receives inhibitory inputs preferentially from specific sectors of nearby SACs that release gamma-aminobutyric acid (GABA) during ND motion and is less likely to connect to other sectors of the same SACs [9,10]. It is worth noting that all of these important findings on the mechanisms of direction selectivity stemmed from an initial observation: SACs and DSGCs have almost completely overlapping stratification patterns [11], allowing them to be potential synaptic partners.

How synaptic inputs contribute to the direction selectivity of DSGCs is a fundamental and important issue [2,3,5]. The GABAergic inhibition from SACs to ON-OFF and ON DSGCs is direction-selective [6,12,13]. But it is less clear if the excitation, which includes glutamatergic and cholinergic components from bipolar cells and SACs, respectively, is also direction-selective [6,12–19], although spatiotemporally offset excitatory inputs have been reported [20–22].

J-RGCs are OFF DSGCs in the mouse retina [23]. They have color opponent responses in the ventral retina [24] and are also orientation-selective to the grating stimulus mediated by the gap-junctional coupling with amacrine cells [25]. Most J-RGCs have highly asymmetric dendritic arbors. The arbor asymmetry is consistent across the retina with somas always displaced to the dorsal side of the dendrites. The PD of J-RGCs aligns with their morphological asymmetry: all J-RGCs prefer the motion in the soma-to-dendrite direction, which is dorsal-to-ventral on the retina and upward motion in the outside world. The dendrites of J-RGCs are restricted in the outermost sub-lamina of the IPL, whereas SAC’s dendrites occupy the intermediate sub-laminas [23,26]. This suggests that J-RGCs do not form extensive synaptic connections with SACs and thus might acquire the direction selectivity from a different source.

We set out to uncover this source by functionally dissecting J-RGCs’ circuit but arrived at a surprising conclusion that the direction selectivity of J-RGCs also depends heavily on the inhibitory inputs from SACs despite their minimal co-stratification in the IPL. Asymmetric connections between J-RGCs and SACs were also observed. Our results identify the synaptic inputs and presynaptic partners of J-RGCs and highlight the critical role of SACs in the emergence of direction selectivity in the mouse retina. The noncanonical connections between J-RGCs and SACs, 2 narrowly stratified neurons in different sub-laminas, also underscore that care should be given when looking for connections between retinal neurons by matching their stratification patterns.

## Results

### Both excitatory and inhibitory synaptic inputs to J-RGCs are direction-selective

**Fig 1:**
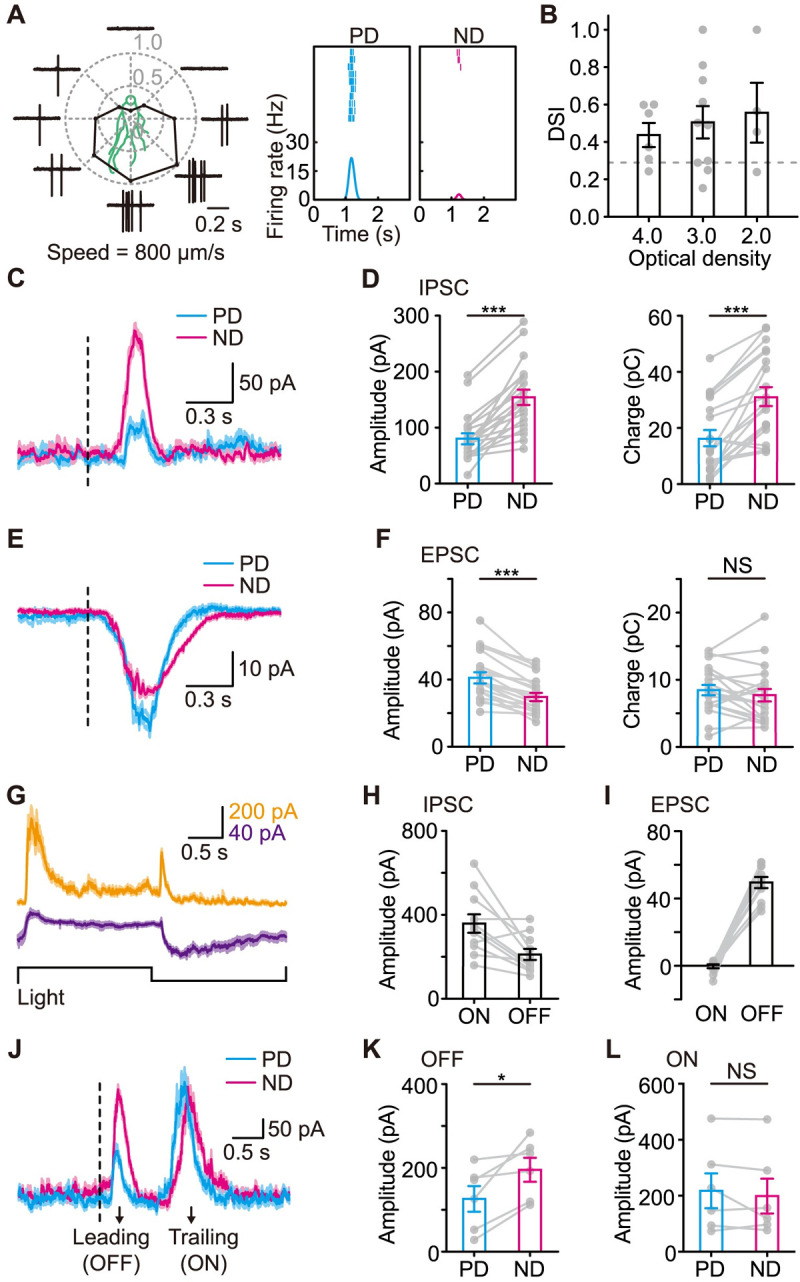
J-RGCs receive DS synaptic inputs during motion stimulus. **(A)** The responses of a J-RGC to a small square black dot moving across its RF center in 8 different directions. The average responses are summarized in the center polar plot. A diagram of the J-RGC’s dendrites is also shown to indicate the correlation between the morphological asymmetry and the preferred motion direction. Right, raster plots and PSTHs of the same J-RGC’s responses to 10 repeats of the PD and ND motion. **(B)** DSI values of J-RGCs’ responses to the moving spot stimulus under different luminance levels (see Methods). Dotted line: DSI = 0.3, threshold for DSGCs. *n* = 6/10/4 cells for optical density = 4.0/3.0/2.0 group. **(C and E)** Representative IPSCs (**C**) and EPSCs (**E**) recorded from a J-RGC during PD and ND motion. Traces are aligned to the estimated time when the leading edge of the moving spot entered the RF center (dotted line). Shaded area around the traces, mean ± SEM, *n* = 10 trials. **(D and F)** Comparison of IPSCs (**D**) and EPSCs (**F**) between PD and ND motion. Left, peak amplitudes; right, total charges. *n* = 20 cells. **(G)** Representative IPSCs (top) and EPSCs (middle) evoked by a spot contrast step stimulus (bottom). Shaded area around the traces, mean ± SEM, *n* = 7 trials. **(H and I)** IPSC (**H**) and EPSC (**I**) amplitudes from the ON and OFF pathways. *n* = 11 cells. **(J)** Representative IPSCs evoked by a black moving bar stimulus. Traces are aligned to the estimated time when the leading edge of the moving bar entered the RF center (dotted line). Shaded area around the traces, mean ± SEM, *n* = 9 trials. **(K and L)** Comparison of IPSC amplitudes from the responses to the leading (**K**) and trailing edge (**L**) between PD and ND motion. *n* = 6 cells. Error bars, SEM. In **D**, **F**, **K**, and **L**, paired *t* test; *, *p* < 0.05; ***, *p* < 0.001; NS, not significant. Data for this figure are in S1 Data. DS, direction-selective; DSGC, direction-selective ganglion cell; DSI, direction selectivity index; EPSC, excitatory postsynaptic current; IPSC, inhibitory postsynaptic current; J-RGC, J-type retinal ganglion cell; ND, null direction; PD, preferred direction; RF, receptive field.

Most J-RGCs have highly asymmetric dendritic arbors. Their PD is the dorsal-to-ventral direction on the retina, which is the same as the soma-to-dendrite direction for J-RGCs (Fig 1A). Since J-RGCs are OFF RGCs with strong surround inhibition [23], a square black dot moving in different directions was used as the motion stimulus for most cases in this study. Such a stimulus elicited typical DS spiking responses from J-RGCs across different light levels (Fig 1A and 1B). To avoid the effects of the color opponent responses, only the J-RGCs located in the dorsal half of the JamB-CreER/Ai9 (RCL-stop-TdTomato) retinas, in which J-RGCs were sparsely labeled, were targeted for recordings. J-RGC labeling by this JamB-CreER line was morphologically and functionally specific as reported [23,27–29]. We recorded the synaptic inputs J-RGCs received during PD and ND motion by whole-cell patch-clamp recordings in the dorsal retina (Fig 1C and 1E). The peak amplitudes of excitatory postsynaptic currents (EPSCs) during PD motion were significantly higher than those during ND motion but the total charges were comparable between PD and ND (Fig 1F), whereas for the inhibitory postsynaptic currents (IPSCs), both the peak amplitudes and total charges were higher during ND motion (Fig 1D). Thus, the direction selectivity of EPSCs and IPSCs is consistent with the overall direction selectivity of J-RGCs. Further, a postsynaptic mechanism is needed for EPSCs to be direction-selective, whereas a presynaptic mechanism likely underlies the direction selectivity of IPSCs.

The excitatory inputs to J-RGCs are from the OFF pathway, and the inhibitory inputs include both ON and OFF components (Fig 1G–1I). We used a black bar moving along its long axis in opposite directions to separate the motion responses into the OFF response to the leading edge and the ON response to the trailing edge. Two peaks were observed from IPSCs during this motion stimulus, an OFF peak followed by an ON peak (Fig 1J). Comparing IPSC responses during PD and ND motion, we found that OFF IPSCs were direction-selective but ON IPSCs were not (Fig 1K and 1L). Together, these data demonstrate that both the excitatory and inhibitory inputs from the OFF pathway are direction-selective.

### Excitatory inputs are spatiotemporally asymmetric

**Fig 2:**
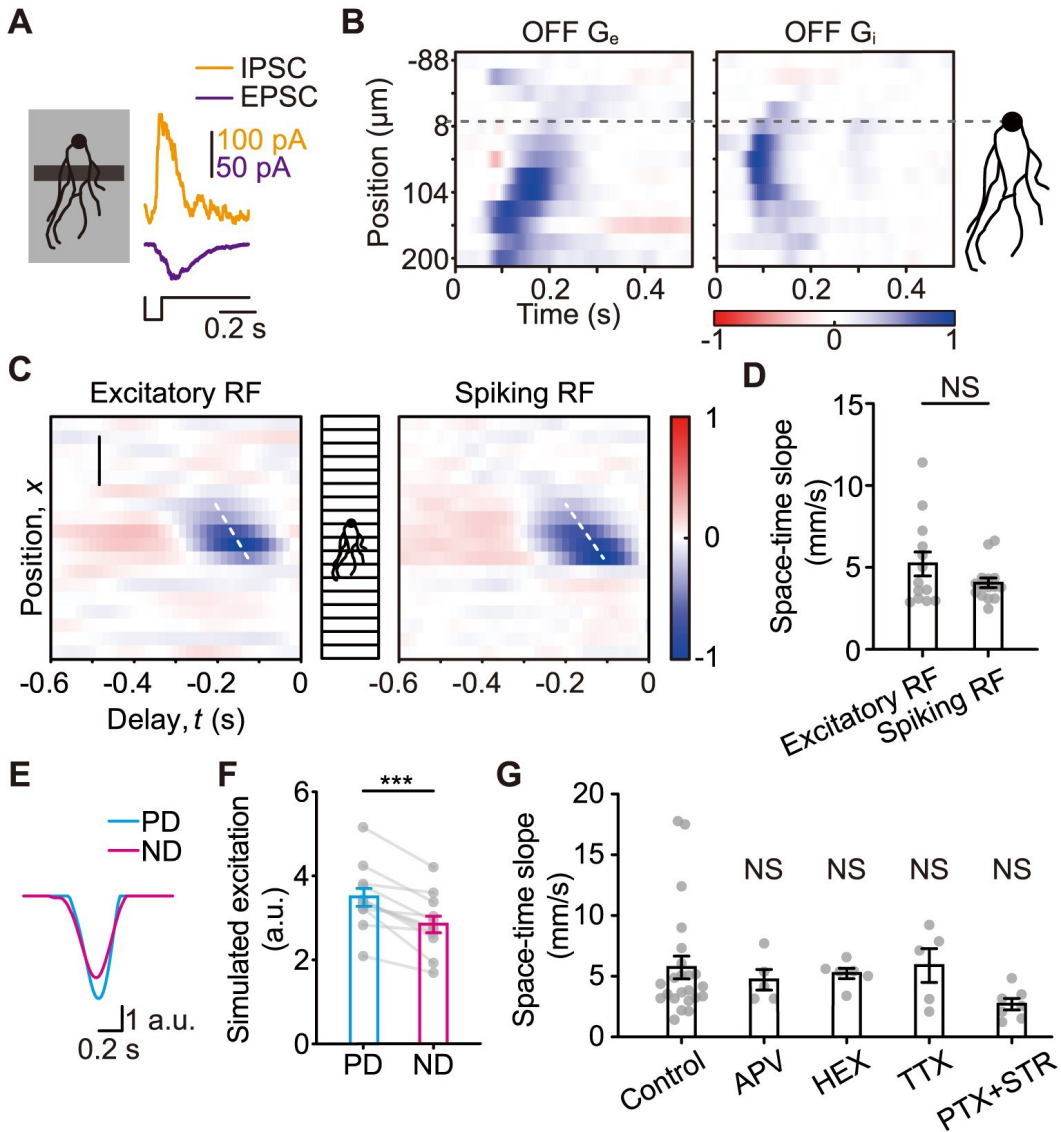
Excitatory synaptic inputs to J-RGCs are spatiotemporally slanted. **(A)** Schematic diagram of the flash bar stimulus used to measure the spatiotemporal properties of the synaptic inputs (left), and representative current traces recorded from a J-RGC when the center of the black bar was 104 μm from the soma (right). The ON and OFF postsynaptic currents were measured separately using white and black flashing bars. The stimulus for the representative responses was a dark flash, thus it elicited OFF responses at the onset. See S1 Fig for the ON response. **(B)** Spatiotemporal profiles of the OFF excitatory (left) and inhibitory (right) conductance responses (G_e_/G_i_) measured using the flash bar stimulus illustrated in **A**. Normalized responses are arranged vertically according to the spatial locations of the flashes, then displayed as a heatmap. White, 0; blue, OFF response. Horizontal, time, 0 is the onset of the flashes; vertical, positions of the flashes, 0 (the dotted line) indicates the center of J-RGC’s soma. **(C)** Representative spiking RF of a J-RGC (right) and the RF for a J-RGC’s excitatory inputs (left). Both RFs were measured using a white noise stimulus with randomly flickering bars along J-RGCs’ PD-ND axis. White, 0; blue, negative, OFF component; red, positive, ON component. Horizontal, time to response; vertical, positions of the bars. Dotted lines, space-time slant in the RF centers. Scale bar, 200 μm. **(D)** Comparison of the space-time slopes in the RF centers between the excitatory and spiking RFs. Unpaired *t* test, 13 excitatory RFs, 15 spiking RFs. **(E)** Simulated excitatory responses to PD and ND motion using a linear model with a J-RGC’s excitatory RF. **(F)** Comparison of the simulated excitatory responses to PD and ND motion. Paired *t* test; ***, *p* < 0.001; *n* = 12 cells. **(G)** Comparison of the space-time slopes of the excitatory RF centers after bath application of NMDA blocker APV, cholinergic blocker HEX, fast sodium channel blocker TTX, or inhibitory blockers PTX+STR to the control condition. Unpaired *t* test, *n* = 23/5/6/5/7 cells for control/APV/HEX/TTX/PTX+STR group. Error bars, SEM. NS, not significant. Data for this figure are in S1 Data. HEX, hexamethonium; J-RGC, J-type retinal ganglion cell; ND, null direction; NMDA, N-Methyl-D-aspartic acid; PD, preferred direction; PTX, picrotoxin; RF, receptive field; STR, strychnine; TTX, tetrodotoxin.

Strategies for the generation of direction selectivity often involve spatial and temporal offsets in different synaptic inputs [2,20]. We therefore studied the spatiotemporal properties of J-RGCs’ synaptic inputs. A small black or white narrow bar quickly flashing at different locations along J-RGCs’ PD-ND axis was used as the stimulus, and the evoked postsynaptic currents were recorded (Figs 2A and S1A). This allowed us to observe the responses from the ON and OFF pathways separately. When the OFF excitatory responses from different locations are aligned temporally to form a spatiotemporal profile, an obvious space-time slant can be observed, indicating faster response to stimulus at the distal parts of the dendritic field and slower response at the proximal parts (Fig 2B, left). In contrast, neither the OFF (Fig 2B, right) nor the ON inhibitory responses (S1B Fig) showed a similar feature. This spatial difference in response latency of excitatory inputs likely contributes to the direction selectivity: PD motion will trigger the slower proximal EPSCs first and the faster distal EPSCs later, resulting in the overlap of these EPSCs at the soma, whereas ND motion will cause the EPSCs to be more spread out over time, and consequently evoke responses with lower peak amplitudes.

We also recorded EPSCs during a white noise stimulus composed of randomly flickering bars and obtained the excitatory receptive field (RF) by reverse correlation. A similar space-time slant was observed (Fig 2C, left). Using this excitatory RF and a simple linear model [30], the DS response of J-RGCs’ EPSCs can be largely predicted (Fig 2E and 2F). Excitatory responses for ND motion are 28% lower than PD motion in actual J-RGCs (Fig 1F, left) and 20% in simulations (Fig 2F). Therefore, the feature in the excitatory RF can explain much of the observed direction selectivity in excitation.

We next examined whether specific types of synaptic transmissions were involved in the generation of this space-time slant in the excitatory RF. Pharmacological blockade of inhibitory inputs with picrotoxin (PTX, GABAergic blocker) and strychnine (STR, glycinergic blocker), N-Methyl-D-aspartic acid (NMDA) inputs with DL-2-Amino-5-phosphonopentanoic acid (APV) or cholinergic inputs with hexamethonium (HEX) in the retina had no effects (Figs 2G and S2A). Voltage-gated sodium channels have been shown to affect the RF of excitatory inputs to ON DSGCs [21], but no similar effects were observed in J-RGCs during the bath of tetrodotoxin (TTX). Gap junction coupling with amacrine cells mainly mediated by connexin36 (Cx36) contributes to J-RGCs’ orientation selectivity [25]. Removing Cx36 selectively in J-RGCs led to overall decreased amplitudes but relatively unchanged direction selectivity of EPSCs during motion stimulus (S2B–S2D Fig, see Discussion). These results indicate that the difference in EPSC response latency does not depend on modulations from the inhibitory pathway, the NMDA pathway, the cholinergic pathway, the voltage-gated sodium channels, or the Cx36-mediated electrical synapses. Excluding these possibilities, the difference in EPSC latency likely comes directly from the connection properties between J-RGCs and bipolar cells: The distal dendrites form connections to bipolar cells with a shorter response latency, whereas the proximal dendrites connect to bipolar cells with a longer latency.

The spatiotemporal offset revealed in the asymmetric structure of J-RGCs’ RFs was reported to be associated with their direction selectivity [23]. Notably, the RFs measured using the spiking response (Fig 2C, right) and EPSCs (Fig 2C, left) are almost indistinguishable from each other (Fig 2D), whereas IPSCs do not share the same spatiotemporal asymmetry as EPSCs (Figs 2B, right and S1B). Do IPSCs contribute significantly to J-RGCs’ direction selectivity?

### Inhibition is critical for direction selectivity

**Fig 3:**
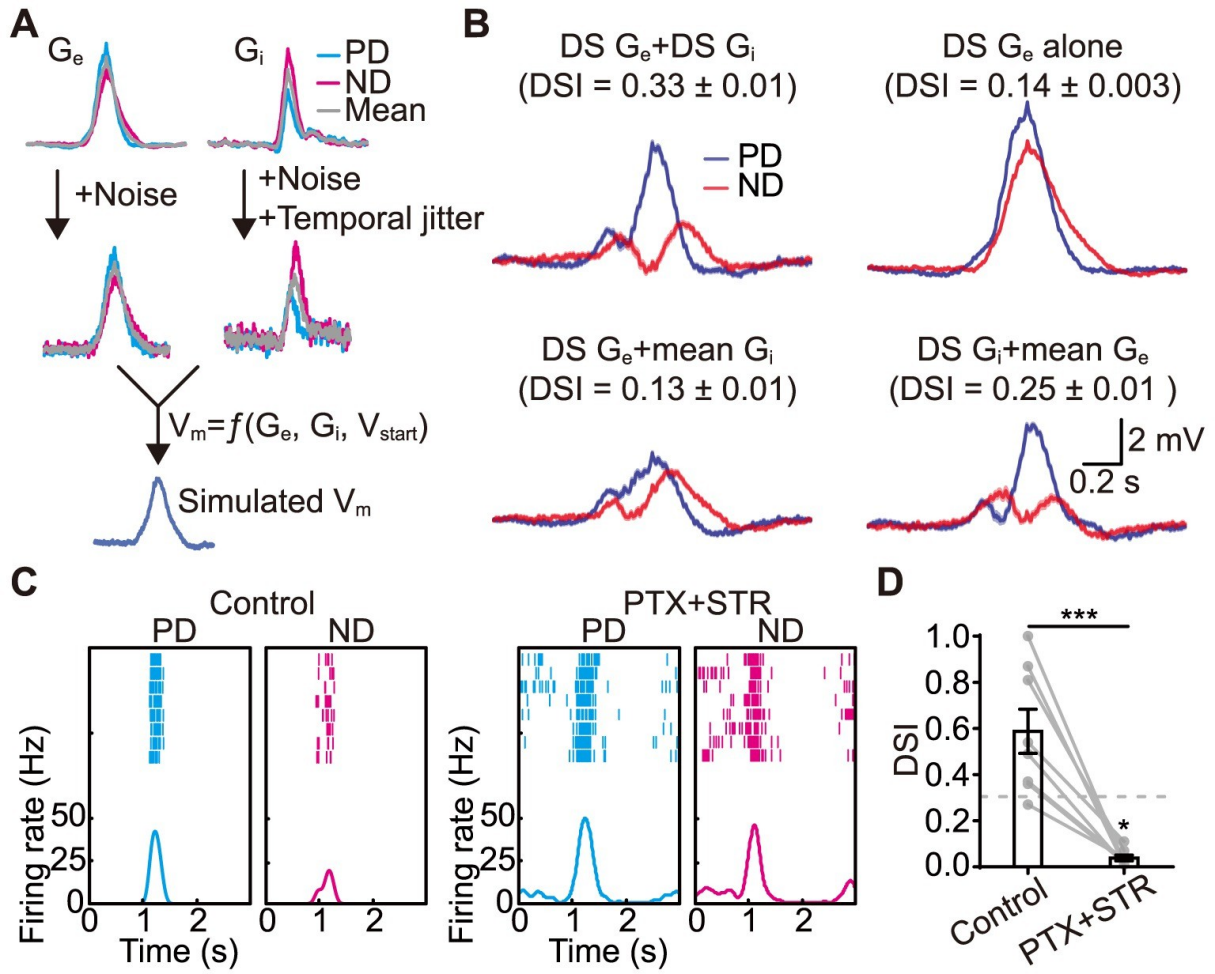
Contributions of excitation and inhibition to J-RGCs’ direction selectivity. **(A)** Simulating J-RGCs’ responses using actual or modified synaptic conductance inputs during motion stimulus. G_e_, excitatory conductance. G_i_, inhibitory conductance. **(B)** Simulated J-RGCs’ responses with actual (DS G_e_+DS G_i_) or modified (the other 3 panels) synaptic inputs when V_start_ = E_inh_+10 mV. See S3 Fig for full results of the simulation. Shaded area around the traces and DSI, mean ± SEM, *n* = 100 trials. **(C)** Spiking responses of a J-RGC to PD and ND motion under control (left) and inhibition blocked condition (right). **(D)** Comparison of DSI values before and after the blockade of inhibition. Dotted line: DSI = 0.3. Control vs. PTX+STR, paired *t* test; ***, *p* < 0.001; *n* = 8 cells. PTX+STR vs. 0, 1 sample *t* test; *, *p* < 0.05. Error bars, SEM. Data for this figure are in S1 Data. DSI, direction selectivity index; J-RGC, J-type retinal ganglion cell; ND, null direction; PD, preferred direction; PTX, picrotoxin; STR, strychnine.

We evaluated the contributions of excitatory and inhibitory inputs to J-RGCs’ direction selectivity using conductance-based integration model [31]. We asked how direction-selective the simulated membrane potential change (ΔV_m_) was when only the neuron’s excitatory or inhibitory inputs were direction-selective (Fig 3A and 3B). Actual synaptic conductance responses recorded from J-RGCs during PD and ND motion were used as the DS inputs, and the averages between PD and ND responses were used as the non-DS inputs. If both the excitation and inhibition were direction-selective, when the resting membrane potential (V_start_) of the cell was between the equilibrium potentials for the inhibitory and excitatory inputs (E_inh_ and E_exc_), direction selectivity of ΔV_m_ could be observed (S3B–S3D Fig). The direction selectivity index (DSI) peaked when V_start_ was moderately above E_inh_. At this membrane potential, DS excitatory and non-DS inhibitory conductance resulted in simulated responses that were much less direction-selective (Fig 3B, bottom left). On the other hand, if the input inhibition was direction-selective but the excitation was identical between PD and ND motion, then the simulated responses were only slightly less direction-selective than control (Fig 3B, bottom right).

We also simulated a condition where the inhibitory inputs were completely removed (Fig 3B, top right). As can be expected, the peak depolarization increased for both the PD and ND motion, but the direction selectivity decreased significantly. We then recorded J-RGCs’ actual responses to the motion stimulus when GABAergic and glycinergic synaptic transmissions in the retina were both blocked with PTX and STR (Figs 3C and S6A and S6D). The average DSI of J-RGCs fell from 0.59 ± 0.10 to 0.04 ± 0.01 (Fig 3D). Thus, as the simulation predicted, removing all inhibition resulted in significantly decreased direction selectivity. It is worth noting, however, that all the recorded cells had slightly stronger PD responses than ND responses without inhibition, resulting in positive DSI values for all cells. We attribute this to the DS excitatory inputs that the cells still received. Together, these results suggest that both excitation and inhibition contribute to J-RGCs’ direction selectivity, with inhibition playing a more critical role.

### DS inhibition originates from a presynaptic source

**Fig 4:**
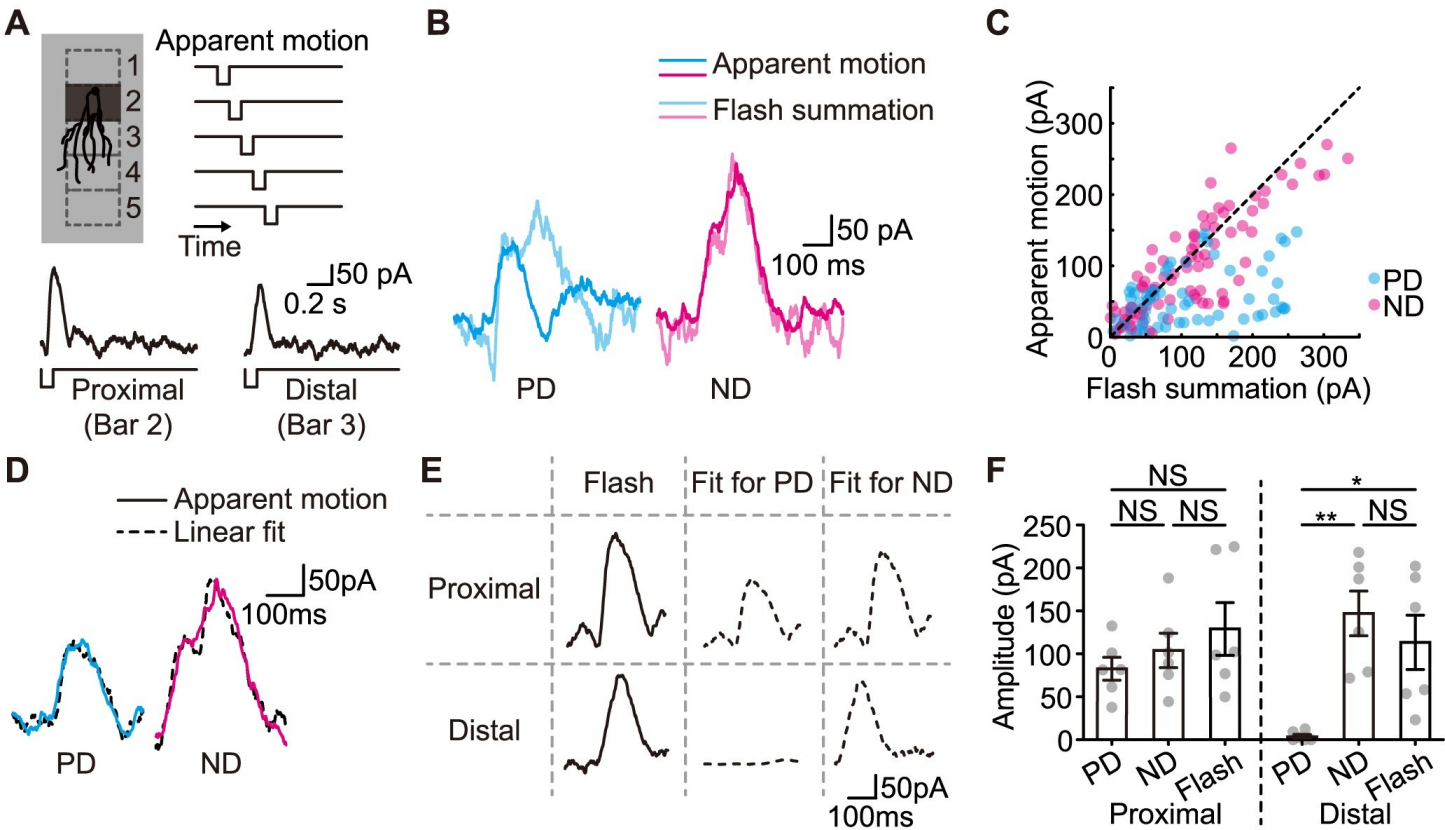
The spatial properties of the DS IPSCs. **(A)** Schematic diagram of the apparent motion stimulus with sequential flashing of bars along the PD-ND axis (top) and the representative IPSC responses to individual proximal (bottom left) and distal (bottom right) bar flashes. Top left: spatial locations of the bars; top right: temporal order of the successive flashes that form the apparent motion stimulus. **(B)** Representative IPSCs evoked by the apparent motion stimulus, compared to the summation of the temporally aligned responses to all component bar flashes recorded in the same J-RGC. **(C)** Comparison of IPSCs evoked by the apparent motion stimulus and the summation results. Each point on the graph compares the mean IPSC amplitudes of a J-RGC’s response in a 20 ms time bin between flash summation (horizontal) and apparent motion (vertical). A total of 6 J-RGCs’ responses are included. Dashed: line of identity. IPSCs during PD apparent motion are significantly lower than the summation of IPSCs evoked by individual flashes. **(D)** Weighted linear combination of the responses to all component bar flashes is used to fit a J-RGC’s apparent motion response. Adjusted R^2^ = 0.92/0.94 for the fitting of the PD/ND responses. This fitting provides an estimate of the contribution of each component bar flash to the total apparent motion response. **(E)** The contributions of the proximal and distal flashes to the PD and ND apparent motion response recorded in an example J-RGC. Flashes at other locations had only minimal contributions to the overall IPSC response. **(F)** Comparison of the peak amplitudes between the IPSC responses to individual bar flashes and the fitted contributions to the PD and ND apparent motion. Only results for the proximal and distal bars are shown. Paired *t* test; *, *p* < 0.05; **, *p* < 0.01; NS, not significant; *n* = 6 cells. Error bars, SEM. Data for this figure are in S1 Data. DS, direction-selective; IPSC, inhibitory postsynaptic current; J-RGC, J-type retinal ganglion cell; ND, null direction; PD, preferred direction.

To better understand the direction selectivity of the inhibition, we recorded inhibitory inputs to J-RGCs under the apparent motion stimulus, a black bar flashing sequentially at adjacent locations with appropriate time intervals to mimic motion (Fig 4A, top) and then compared the IPSC response to the linear combination of the separately measured responses to individual bar flashes. We adjusted the flash duration and size of the bars so that individual bar flashes elicited strong enough response in J-RGCs (S4 Fig), and the apparent motion stimulus composed of these flashing bars elicited responses that were indistinguishable from the responses to a smooth motion stimulus at the same speed (S5 Fig). Only within a limited region, slightly smaller than a J-RGC’s dendritic field, did we see significant IPSC responses to individual flashing bars (Figs 4A, bottom and S4). This region was generally covered by 2 bars of the stimulus: the proximal bar covering the soma and the proximal dendrites, and the distal bar covering the intermediate part of the dendritic field.

We first tested if the recorded IPSCs during the apparent motion stimulus were equal to the summation of the responses to the individual bar flashes. It was indeed the case for the response to ND motion (Fig 4B, right and 4C, magenta), but the response to PD motion was significantly weaker than the summation (Fig 4B, left and 4C, cyan). During the PD apparent motion, the proximal bar flashed first and was immediately followed by the distal flash. Looking at the current traces more closely, the response to the PD motion resembled the response to the proximal flash alone. This suggests that the response to the distal flash was severely suppressed by the proximal flash a short time ago.

We then quantified this effect. The response of each J-RGC to the apparent motion stimulus was well fitted to a weighted linear combination of the responses from the same J-RGC to individual bar flashes (Fig 4D). The contribution of each bar flash to the overall response during the apparent motion was thus represented by the product of its fitting coefficient and the individual flash response (Fig 4E). For ND motion, the contributions from the proximal and distal bars were both similar to the responses evoked by each individual bar flash. For PD motion, on the other hand, the distal bar made close to zero contribution (Fig 4F). Contributions from other bar flashes were always around baseline and are therefore not compared (S4 Fig).

These results corroborate the observation that the total amount of inhibition is lower during PD motion than ND motion (Fig 1C) and suggest that the inhibitory neurons for these DS IPSCs should be direction-selective themselves. The current results are also reminiscent of feed forward inhibition: The response elicited by the proximal bar flash likely feed forward to inhibit the response to the distal bar flash during the apparent stimulus. SACs are the only DS inhibitory retinal neurons discovered so far, which form feedforward inhibitory networks among themselves [32]. The inhibitory inputs from SACs to ON-OFF DSGCs also show similar response patterns to the apparent motion stimulus [12]. Thus, we started to contemplate SACs as the candidates for J-RGCs’ inhibitory presynaptic partners despite their distinct stratification patterns in the IPL.

### SACs form inhibitory connections to J-RGCs

**Fig 5:**
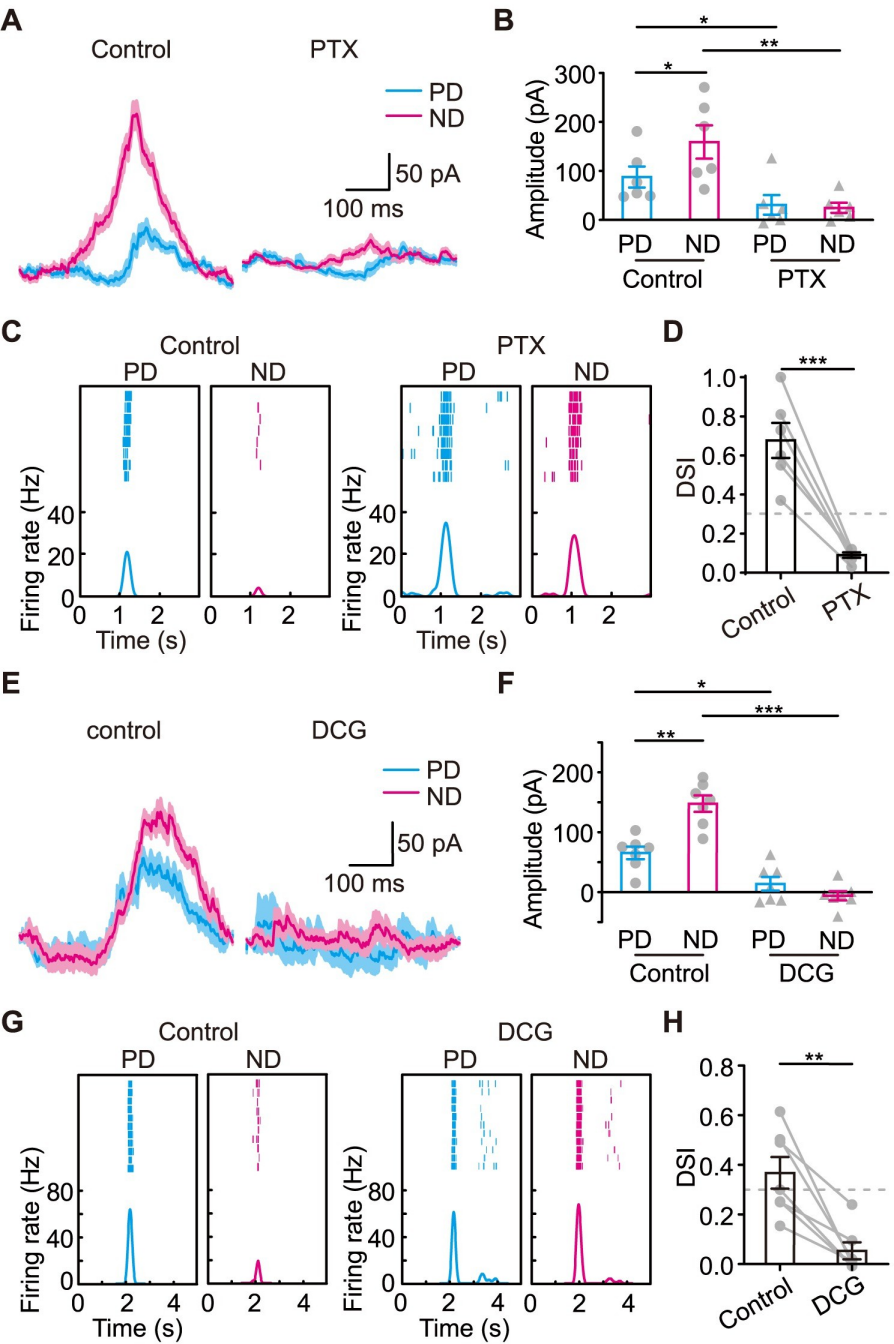
The DS IPSCs depend on SACs. **(A)** Representative OFF IPSCs recorded during PD and ND motion before (left) and after (right) bath application of PTX to block the GABAergic inhibition. **(B)** Summary of the effect of PTX on the peak amplitudes of motion evoked OFF IPSCs. *n* = 6 cells. **(C)** The spiking response of a J-RGC to PD and ND motion before (left) and after (right) bath application of PTX. **(D)** Comparison of DSI values before and after bath application of PTX. *n* = 6 cells. **(E)** Representative OFF IPSCs recorded during PD and ND motion before (left) and after (right) bath application of DCG-IV to block SACs’ outputs. **(F)** Summary of the effect of DCG-IV on the peak amplitudes of motion evoked OFF IPSCs. *n* = 7 cells. **(G)** The spiking response of a J-RGC to PD and ND motion before (left) and after (right) bath application of DCG-IV. **(H)** Comparison of DSI values before and after bath application of DCG-IV. *n* = 7 cells. Error bars, SEM. Dotted lines in **D** and **H**: DSI = 0.3. In **A** and **E**, shaded area around the traces, mean ± SEM, *n* = 16 trials. In **B**, **D**, **F**, and **H**, paired *t* test; *, *p* < 0.05; **, *p* < 0.01; ***, *p* < 0.001. Data for this figure are in S1 Data. DS, direction-selective; DSI, direction selectivity index; GABA, gamma-aminobutyric acid; IPSC, inhibitory postsynaptic current; J-RGC, J-type retinal ganglion cell; ND, null direction; PD, preferred direction; PTX, picrotoxin; SAC, starburst amacrine cell.

We tested this hypothesis pharmacologically using PTX and (2*S*,2′*R*,3′*R*)-2-(2′,3′-Dicarboxycyclopropyl)glycine (DCG-IV) first. PTX blocks the GABAergic inhibitory pathways including those of SACs’. DCG-IV is a type 2 metabotropic glutamate receptor agonist, which specifically blocks all the outputs of SACs in the retina [33,34]. OFF IPSCs to J-RGCs during motion were eliminated under either PTX (Fig 5A and 5B) or DCG-IV (Fig 5E and 5F). And J-RGCs’ spiking responses to motion were no longer direction-selective (Figs 5C, 5D, 5G, 5H, S6A–S6C and S6E). The results suggest that GABAergic inhibition from SACs is critical for J-RGCs’ direction selectivity.

**Fig 6:**
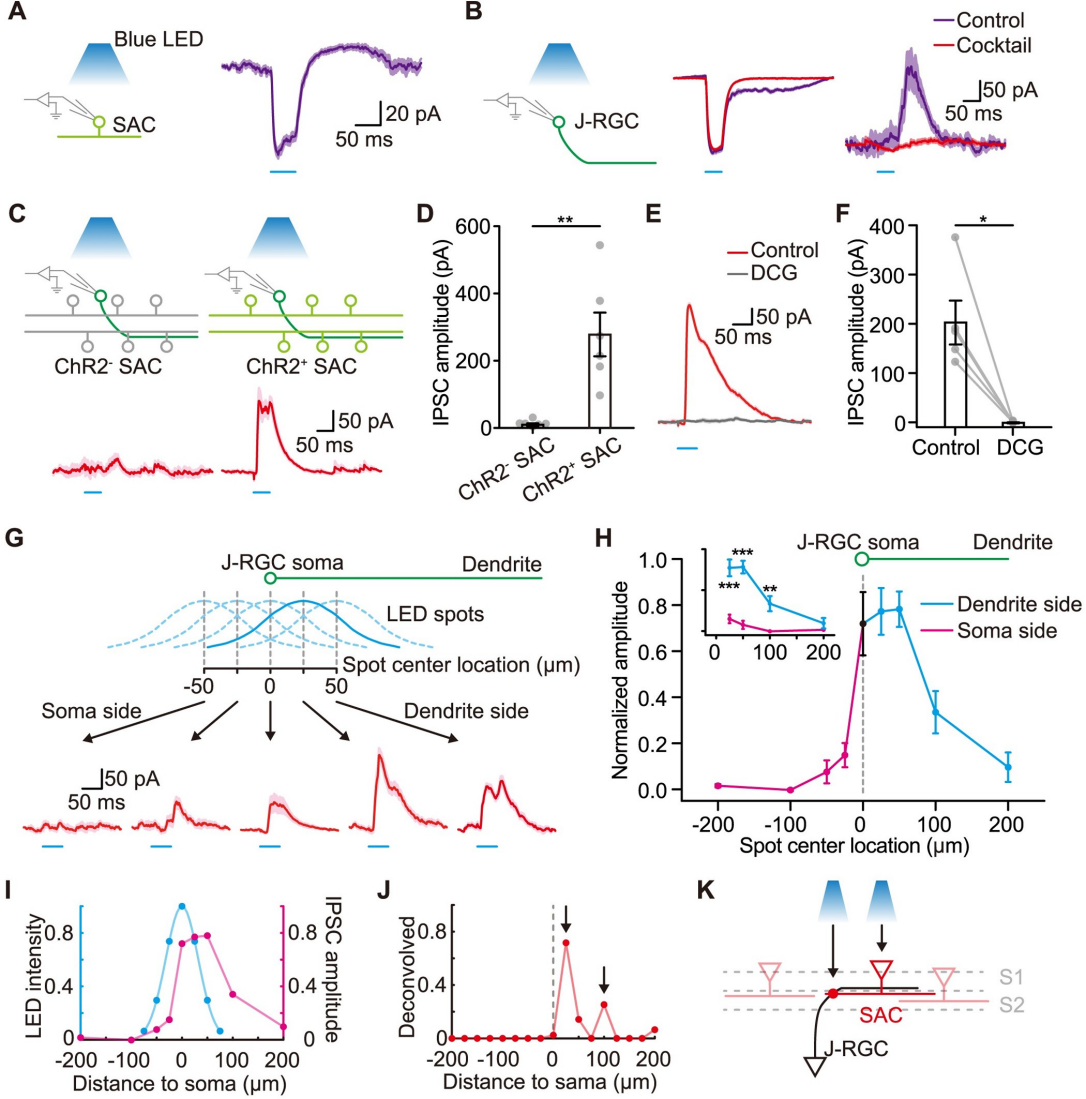
Asymmetric connections between J-RGCs and SACs. **(A)** Optogenetic activation of ChR2^+^ SACs (left) and the representative photocurrents recorded from a SAC (right, holding potential −67 mV). **(B)** Optogenetic activation of ChR2^+^ J-RGCs (left) and the representative photocurrents (middle, holding potential −67 mV) and IPSCs (right, holding potential 0 mV) recorded from a J-RGC under the control condition (purple) or the drug cocktail condition (red). The drug cocktail contained APV, CNQX, STR, and HEX to block non-GABAergic synaptic transmissions in the retina. The same cocktail was used in all the following optogenetic experiments in **C**–**H**. **(C)** Representative IPSCs recorded from J-RGCs, evoked by blue LED pulse flashes with ChR2^-^ (left) and ChR2^+^ (right) SACs in the retina. **(D)** Comparison of the peak amplitudes of IPSCs recorded from J-RGCs with ChR2^−^ and ChR2^+^ SACs. Unpaired *t* test; **, *p* < 0.01; *n* = 6 cells for each group. **(E)** Representative optogenetically evoked IPSCs recorded in a J-RGC before and after bath application of DCG-IV. **(F)** Summary of optogenetically evoked IPSC peak amplitudes before and after bath application of DCG-IV. Paired *t* test; *, *p* < 0.05; *n* = 5 cells. **(G)** Experimental diagram for mapping the inputs from SACs (top) and representative results (bottom). The experiment maps the spatial distribution of SACs that provide inhibitory inputs to a J-RGC: Small spots of LED light pulse were positioned along the J-RGC’s PD-ND axis at different distances from its soma and evoked IPSCs recorded in the J-RGC. The spots are represented by their center locations and the distribution of light intensity within each spot (approximated by the cyan gaussian curves, the solid curve indicating the spot that evoked the strongest IPSCs). The full size of the J-RGC’s dendritic field along the PD-ND axis is shown on top for comparison. Example IPSC traces evoked by the LED spots at several locations are shown to highlight the spatial asymmetry around the soma. **(H)** Summary of the input strength from SACs along J-RGCs’ PD-ND axis. The normalized peak amplitudes of IPSCs are plotted against the locations of the spot centers. Distance > 0: on the dendrite side; distance < 0: on the side without dendrites (soma side). Inset: comparison of IPSC peak amplitudes from spots located equal distances away but on opposite sides of the soma. Unpaired *t* test; **, *p* < 0.01; ***, *p* < 0.001; *n* = 5/5/4/5/6/5/5/6/5 cells at distances −200/−100/−50/−25/0/25/50/100/200 μm. **(I)** The measured input strength from SACs along J-RGCs’ PD-ND axis (magenta, same as in **H**) and the spatial distribution of relative intensity for the LED spot used for the measurement (cyan). **(J)** Hotspots (black arrows) of SAC inputs revealed by deconvolution using the inputs in **I**. **(K)** Schematic diagram to illustrate the results in **J**. The 2 black arrows indicate the locations of J-SAC synapses and the upstream SAC soma. LED light at these locations can elicit IPSCs in J-RGCs much more efficiently than that at other locations. S1/S2, sub-lamina 1/2 of the IPL. Error bars, SEM. In **A**–**C**, **E**, and **G**, shaded area around the traces, mean ± SEM, *n* = 10 trials. Data for this figure are in S1 Data. GABA, gamma-aminobutyric acid; HEX, hexamethonium; IPSC, inhibitory postsynaptic current; J-RGC, J-type retinal ganglion cell; SAC, starburst amacrine cell; STR, strychnine.

We further tested the SAC to J-RGC connections directly by optogenetically activating SACs and recording evoked IPSCs in J-RGCs using JamB-CreER/ChAT-Cre/Ai32 mice. The ChAT-Cre transgene was used to drive the expression of channelrhodopsin-2 (ChR2) in SACs [19,34–37]. Two-photon imaging was used to identify J-RGCs morphologically for recording. As ChR2 was also expressed in J-RGCs, we needed to ensure that we could isolate the ChR2-mediated SAC-IPSCs from direct ChR2 photocurrents in J-RGCs first. We examined the effect of ChR2 activation in SACs alone and J-RGCs alone using ChAT-Cre/Ai32 and JamB-CreER/Ai32 mice (Fig 6A and 6B). ChR2 activation by 50 ms pulses of 470 nm LED light evoked substantial photocurrents immediately in SACs and J-RGCs at −67 mV. A drug cocktail of STR, HEX, APV, and 6-Cyano-7-nitroquinoxaline-2,3-dione (CNQX) was used to block all the excitatory and glycinergic inhibitory transmissions, removing regular light responses of the retina, and leaving only GABAergic synaptic connections intact. The recorded currents at 0 mV under this drug cocktail were flat, thus holding cells at 0 mV in the following experiments can effectively isolate the IPSCs a J-RGC receives from the interference of ChR2 photocurrents in the same J-RGC.

We then activated ChR2 in JamB-CreER/ChAT-Cre/Ai32 retinas under the same drug cocktail. Significant IPSCs could be reliably recorded from J-RGCs when ChR2 was activated in SACs. No IPSCs were observed in control retinas where ChR2 was not expressed in SACs (Fig 6C and 6D). Moreover, DCG-IV completely eliminated these IPSCs (Fig 6E and 6F). Since SACs were the only inhibitory neurons expressing ChR2 in these retinas (see Methods), our results prove that SAC activation can evoke GABAergic IPSCs in J-RGCs.

The drug cocktail blocked all the non-GABAergic transmissions, therefore, the connections between J-RGCs and SACs are either direct (monosynaptic) or via serial GABAergic connections. Under our experimental setup, the latency of the optogenetically evoked photocurrents recorded in SACs (18.5 ± 4.2 ms to peak, *n* = 4 cells) corresponds well with the latency of the IPSCs recorded in J-RGCs (23.6 ± 4.4 ms to peak, *n* = 9 cells) and their difference is within the range of monosynaptic transmission [36,38]. Thus, the connections between J-RGCs and SACs are likely monosynaptic.

### Connections between J-RGCs and SACs are spatially asymmetric

The direction selectivity of ON-OFF DSGCs relies upon the highly selective and asymmetric connections with SACs. We asked if similar asymmetric connections exist between J-RGCs and SACs. We flashed a 470 nm LED light spot at different locations along J-RGCs’ PD-ND axis to activate SACs locally and then recorded the evoked IPSCs in J-RGCs. Only spot flashes on the dendrite side of J-RGCs evoked significant IPSCs (Fig 6G and 6H). This is qualitatively consistent with the spatial profile of the OFF inhibitory conductance which is largely restricted to the proximal half of the dendritic field (Fig 2B, right).

**Fig 7:**
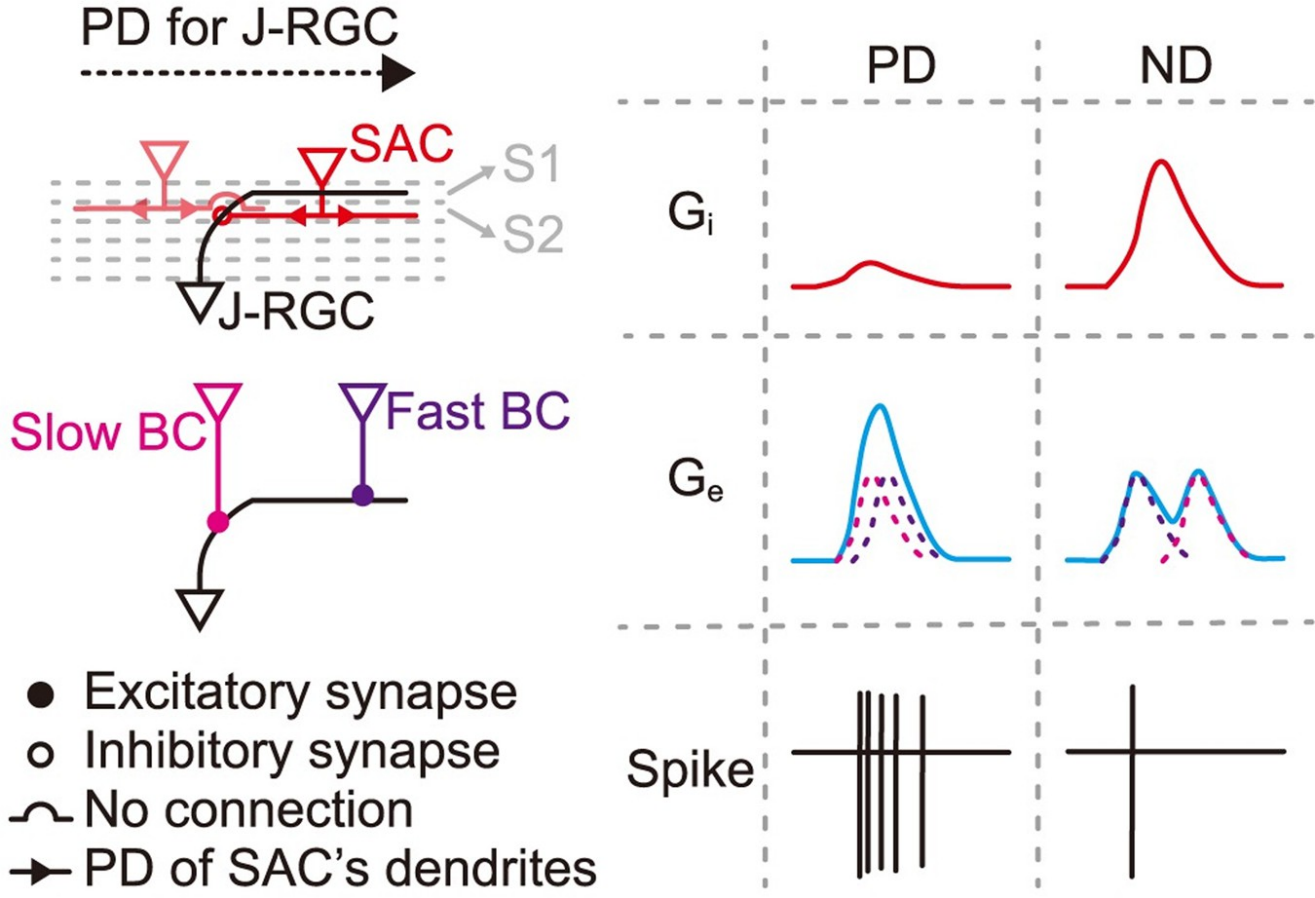
A working model for the generation of J-RGCs’ direction selectivity. Inhibition: only SACs on the dendrite side of J-RGCs make synaptic connections. PDs of different SAC sectors are indicated by the red arrow heads. Excitation: slow bipolar cells connect to the proximal dendrites of J-RGCs, while fast bipolar cells connect to the distal dendrites, resulting in more temporal overlap of excitatory inputs for PD motion, and less overlap thus less summation during ND motion. S1/S2, sub-lamina 1/2 of the IPL. BC, bipolar cell. G_e_, excitatory conductance. G_i_, inhibitory conductance. IPL, inner plexiform layer; J-RGC, J-type retinal ganglion cell; ND, null direction; PD, preferred direction; SAC, starburst amacrine cell.

However, the optogenetically measured map of SAC inputs (Fig 6H) does not directly correspond to the actual locations of presynaptic SACs for 2 reasons: (1) the LED light spot had a large point spread function; and (2) ChR2 was expressed in both the synaptic terminals and the somas of SACs, thus activation at either site could trigger IPSCs to J-RGCs. We then used deconvolution to remove the smearing effect caused by the size of the light spot (Figs 6I and 6J and S7) to reveal that stimulation of SACs at 2 zones was especially effective in eliciting IPSCs in J-RGCs: about 100 μm away from J-RGC’s soma on its dendrite side and a narrow region close to J-RGC’s soma (Figs 6J and S7G). Considering the size of the SAC arbor and the locations of their output synapses [39], the former is likely where the somas of the upstream SACs are located, and the latter corresponds well with the only location where J-RGC and SAC dendrites meet and have potential to form synapses (Fig 6K). Thus, the actual site of the synaptic connection from SACs to J-RGCs is likely restricted to a narrow region close to J-RGC’s soma and not distributed throughout its dendritic field. Incidentally, J-RGCs have denser inhibitory synapses on the proximal dendrites than on the distal dendrites [27], consistent with this supposition. Furthermore, among all the SACs in the vicinity of a J-RGC’s soma, only those on J-RGC’s dendrite side actually make connections (Fig 7, also see Figs 2B, right and S4). Therefore, the connections between J-RGCs and SACs are highly specific and asymmetric.

## Discussion

In this report, we found that both excitation and inhibition to J-RGCs were direction-selective. Simulation indicated that DS inhibition was critical for J-RGCs’ direction selectivity, and blocking inhibition indeed severely impaired it. Pharmacological and optogenetic experiments then showed that SACs were the source of this DS inhibition and that SAC to J-RGC connections were spatially asymmetric. These findings not only reiterate the critical and perhaps pervasive role of SACs in the emergence of direction selectivity in the retina, but also highlight the complexity of retinal circuit formation beyond the potential restriction imposed by retinal neurons’ stratification patterns.

Since J-RGCs’ dendrites do not co-stratify with the dendrites of SACs’, the likelihood of them making functionally meaningful connections had been considered low [23]. Nevertheless, virus tracing results showed that an RGC with J-RGC like morphology was one of SACs’ postsynaptic targets [40]. And our results here demonstrate functional synaptic connections between J-RGCs and SACs for the first time, to our knowledge. The most likely location for their synapses is the narrow region where the ascending J-RGC dendrites pass through the dense SAC stratification layer. Direct observation of the asymmetric J-SAC connections is still needed to confirm our findings; nevertheless, the result so far reminds that the passerby processes deserve more attention when considering circuit connections between retinal neurons. A similar phenomenon has also been observed within the circuit of M1 ipRGCs, which stratify in the outer IPL where the OFF bipolar cells arborize but receive excitatory inputs directly from the ON bipolar cells [41,42]. Therefore, J-RGCs are not the only cell type that breaks the established rules of retinal stratification and circuits.

The connection between SACs and J-RGCs was observed in the presence of a drug cocktail to block all the non-GABAergic transmissions. Together with 5 ms latency between SAC activation and J-RGC responses, a direct GABAergic connection is strongly suggested. SACs use both GABA and acetylcholine as transmitters, and the drug cocktail contained HEX to block cholinergic transmissions [15,19,38]. A recent report showed that 100 μm of HEX was unable to block α7-nicotinic acetylcholine receptors (nAChRs) on several types of bipolar cells [43]. Even though we used a higher concentration of HEX (300 μm) sufficient to block most of the α7-nAChR currents in vitro [44], caution needs to be taken in completely ruling out the involvement of cholinergic transmissions between SACs and J-RGCs. J-RGCs also receive inhibitory inputs from other amacrine cells besides SACs, including glycinergic amacrine cells [25]. The inhibition from the ON pathway, which is not direction selective, was also impacted by the blocking of SACs’ outputs. Thus, SACs may also be indirectly involved in other aspects of J-RGCs’ functions.

The excitatory inputs also contribute to J-RGCs’ direction selectivity (Figs 3B and S3), especially during high speed motion (S8 Fig). Since the total excitation remains the same between PD and ND motion, and no pharmacological means were able to remove the space-time slant in the excitatory inputs, this feature likely exists in the excitatory connections themselves. Bipolar cells with different response latencies connecting with different parts of J-RGC’s dendrites readily explain the observed space-time slant (Fig 7). This proposal is consistent with a previous morphological study suggesting that J-RGCs make selective connections to subtypes of bipolar cells [45]. Based on the response latency and stratification depth of different bipolar cells [46], long-latency type 4 bipolar cells likely connect to J-RGC’s ascending proximal dendrites, and short-latency type 1 bipolar cells are good candidates for connecting to J-RGC’s distal dendrites. This mechanism for direction selectivity is not unique to J-RGCs: both ON DSGCs and SACs have been shown to possess a similar space-time slant in their excitatory RFs [21,47], and SACs make selective connections with subtypes of bipolar cells along SACs’ dendrites [22]. Together, these results suggest a common theme for direction selectivity in the excitatory pathway.

J-RGCs have been reported to completely lack excitatory currents with their spiking patterns dominantly modulated by inhibition and outward currents from electrical synapses [25]. But we have recorded synaptic currents with a reversal potential of 10 mV after pharmacologically blocking inhibitory synaptic inputs (S9 Fig) during visual stimuli. Other reports have also suggested the connections between J-RGCs and bipolar cells by morphology [45] and function [24]. The predominant (possibly the only) subtype of connexins for gap junctions between J-RGCs and amacrine cells have been suggested to be Cx36 [25], and the Cx36^f/f^ mice have been successfully used to knock out Cx36 in retinal neurons [48,49]. However, after we used it to remove the Cx36-mediated gap junction coupling in J-RGCs, the excitatory inputs were still direction-selective, indicating that the direction selectivity of excitation is not fully dependent on the electrical synapses. Nevertheless, the peak EPSC amplitude did decrease noticeably, highlighting the significance of gap junction coupling in J-RGCs’ function. The exact contributions of gap junction coupling (especially from other subtypes of connexins) in the direction selectivity of J-RGCs need further study with better pharmacological or transgenic tools in the future.

Besides inheriting direction selectivity from inhibitory and excitatory pathways, ON-OFF DSGCs also employ other mechanisms, such as E:I spatiotemporal offsets or local interactions in the dendrites, dendritic spikes, and dendro-dendritic excitation [37,50–53]. Our simulation results showed that biased inhibition was sufficient to create the direction selectivity observed in J-RGCs, but does not exclude the possibility that these other mechanisms may also contribute.

In addition to being direction-selective, J-RGCs are also orientation-selective [25] and involved in color vision [24]. It remains unanswered what roles they perform in mouse visual perception and behavior with such a complex set of functions. Differences in visual stimuli used by these various studies might account for these diverse observations and J-RGCs might be able to extract different visual features in different visual environments. In this report, we focused our study on the direction selectivity of J-RGCs. To this end, we recorded only the J-RGCs in the dorsal retina to avoid potential interaction with color vision in the ventral retina and only used a dark moving dot as the motion stimulus (instead of moving bars and gratings) to avoid confounding the DS response with an orientation-selective component. Other stimulus dimensions such as speed, color, intensity, contrast, and background luminance level were fixed or limited in a narrow range comparable to what is typically used to study ON-OFF and ON DSGCs. Under such conditions, most of the recorded J-RGCs had DSI above 0.3, thus were direction selective by most standards [47,54–56]. The interplay between this direction selectivity and other aspects of J-RGCs’ response properties under more diverse visual environments requires further investigation.

Asymmetric functional connections between ON-OFF DSGCs and SACs have been observed [10,35], but the mechanisms underlying the formation of these highly selective connections are still elusive. We now show that J-RGCs also form similarly selective connections with SACs. Whether the same mechanisms are involved remains to be seen. The exploration of the similarities and differences between SAC to J-RGC and SAC to ON-OFF DSGC connections may help solve this issue.

## Materials and methods

### Ethics statement

All the experimental procedures were approved by the Animal Care and Use Committee of Center for Excellence in Brain Science and Intelligence Technology, Chinese Academy of Sciences (approval number: NA-012-2019) and were performed according to the Guideline for Ethical Review of Animal Welfare (GB/T 35892–2018) and the General Requirements for Animal Experiments (GB/T 35823–2018) of the People’s Republic of China.

### Animals

Mice were housed in the 12/12 h light/dark specific-pathogen-free environment. JamB-CreER mice were kindly provided by Joshua Sanes. They were crossed with the reporter line Ai9 (Jackson Lab # 007909) for targeted recording. In the optogenetic experiments, triple positive mice of JamB-CreER, ChAT-Cre (Jackson Lab # 006410) and Ai32 (Jackson Lab # 024109) mice were used. To remove Cx36 selectively in J-RGCs, JamB-CreER/Cx36^f/+^ and Ai9/Cx36^f/+^ were crossed together (Cx36^f/f^ from EMMA # 02510). Tamoxifen (Sigma, 50 to 100 μg in sunflower seed oil) was injected intraperitoneally 3 times at P1/2/4 for the induction of CreER. Both male and female adult mice (1 to 6 months old) were used in our study.

### Electrophysiology

Mice were dark-adapted for over 2 h before sacrificed under dim red light. The retinas were isolated under infrared light and then put into oxygenated Ringer’s solution (110 mM NaCl, 1 mM CaCl_2_, 2.5 mM KCl, 1.6 mM MgCl_2_, 22 mM NaHCO_3_, 10 mM glucose) at room temperature. The dorsal halves of the retinas were used for recordings. For cell-attached recordings, both the extracellular and pipette solutions were the Ringer’s solution. For whole-cell recordings, pipettes were filled with an intracellular solution (120 mM Cs methanesulfonate, 5 mM NaCl, 10 mM HEPES, 5 mM EGTA, 5 mM QX314, 0.5 mM CaCl_2_, 4 mM ATP, 0.5 mM GTP) and the retinas were superfused with artificial cerebrospinal fluid (126 mM NaCl, 26 mM NaHCO_3_, 2.5 mM KCl, 2 mM CaCl_2_, 2 mM MgCl_2_, 1.25 mM NaH_2_PO_4_, 10 mM glucose). To isolate the excitatory and inhibitory synaptic inputs, cells were held at the reversal potential of inhibition (−67 mV) and excitation (0 mV). Series resistance was not compensated and only cells with series resistances less than 20 MΩ were used. Signals were acquired with MultiClamp 700B amplifier (Molecular Devices), digitized at 10 kHz and low-pass filtered at 1 kHz.

J-RGCs’ visual responses were recorded as described previously [23]. J-RGCs were identified in the JamB-CreER/Ai9 retinas by their expression of TdTomato protein using brief excitations (about 50 ms) with a lime LED (567 nm, Rebel LED, Lumileds).

To record optogenetically evoked response, J-RGCs and SACs were targeted with 2-photon illumination (960 nm, Chameleon, Coherent). The JamB-CreER/ChAT-Cre/Ai32 retinas were carefully characterized first. Cre-mediated expression could be induced occasionally in some bipolar and non-SAC amacrine cells, possibly due to weak expression of JamB-CreER in these cells. Whole-cell recordings were performed only from the retinas without non-SAC amacrine cells labeled. All the EYFP positive amacrine cells in the recorded retinas were confirmed to be ChAT-positive SACs by postrecording immunostaining.

ChR2 was activated using a high-power LED (470 nm, Rebel LED, Lumileds) through the objective lens with the mean light intensity of 2.4 mW/mm^2^ within a spot of about 108 μm diameter, the location of which was positioned by the computer-controlled stage; 12.5 μm PTX (Tocris), 0.25 μm STR, 10 μm DCG-IV (Tocris), 300 μm HEX, 50 μm APV, 10 μm CNQX, and 1 μm TTX (Fisheries Technology Development Company, Hebei, China) were used for the pharmacological experiments. All drugs were purchased from Sigma except where noted.

### Visual stimuli

Visual stimuli, generated with a custom program and delivered from a computer-driven Acer K130 projector, were focused onto the photoreceptors through the custom-made microscope condenser at a scale of 8 μm/pixel and a frame rate of 60 Hz. White light was used, and the average intensity for all the stimuli under the neutral density filter (Thorlabs) with optical density at 3.0 was equivalent to the following photon flux values for the 3 mouse photoreceptors: rod, 5.9 × 10^4^ photons/s/μm^2^ at 500 nm; M cone, 6.7 × 10^4^ photons/s/μm^2^ at 511 nm; S cone, 3.9 × 10^2^ photons/s/μm^2^ at 370 nm. Illuminance levels were modulated in Figs 1B and S6F by different neutral density filters with optical density at 2.0 and 4.0.

The RF centers of the recorded J-RGCs were determined by a manually controlled small flashing spot near the somas and were used to center all the subsequent stimuli. To measure the direction selectivity, a square black dot (160 × 160 μm) moving at 800 μm/s (except the S8 Fig where 2,000 μm/s was also used) was used, in 8 different directions for cell-attached recordings and 2 opposite directions along the PD-ND axis for whole-cell recordings. The light step stimulus consisted of a spot of 160 μm in diameter flashing between white and black at 0.25 Hz. A black bar (800 × 160 μm) moving at 800 μm/s along its long axis enabled the clear segregation of the OFF responses to the leading edge and ON responses to the trailing edge. A white or black bar (160 × 80 μm, 0.1 s duration, 2 s interval) pseudorandomly appearing at 13 locations at 24 μm spatial interval along the PD-ND axis was used to map the spatiotemporal profile of the ON and OFF synaptic inputs. The stimulus for excitatory/spiking RF mapping was composed of 24 pseudorandomly flickering black or white bars (120 × 56 μm) at 60/20 Hz for 180 s/15 min. The apparent motion stimulus presented a black bar (160 × 80 μm, 0.1 s duration) flashing sequentially at 7 adjacent locations 80 μm apart. The corresponding smooth motion stimulus presented a moving bar of the same size along the same trajectory at the equivalent speed of 800 μm/s. The individual bar flash stimulus for decomposion was similar to the apparent motion stimulus except that the order of flashes was pseudorandom and there were 2 s intervals between flashes.

### Modeling

To simulate the membrane potential responses to motion under different conditions, a conductance-based integration model [31] was used as the difference equation below:

$$V_{m}(t+1)\;\;=\;\;-\frac{dt}{C}\left\{G_{e}(t)\left[V_{m}\;(t)-E_{exc\;}\right]+G_{i}(t)\left[V_{m}(t)-E_{inh\;}\right]+G_{rest\;}\left[V_{m}(t)-V_{start\;}\right]\right\}+V_{m}(t),$$ (1)

where E_exc_ = 0 mV, E_inh_ = −67 mV, and the cell capacitance C (3.96 nF) and the resting conductance G_rest_ (3.60 nS) were the average values from 8 recorded J-RGCs. G_e_ and G_i_ from the same 8 J-RGCs were calculated as describe previously [13], aligned to the peak and averaged separately to get the mean PD G_e_, PD G_i_, ND G_e_, and ND G_i_. Gaussian noise and temporal offset matching the actual level of the noise in the recorded conductance were then added and the results were taken as the input G_e_ and G_i_ to the equation. Different resting membrane potential (V_start_) values (from −65 mV to −40 mV at a step of 5 mV) were tested.

### Analysis and statistics

The vector sum magnitude, the spiking and excitatory RF, and the simulation of the excitatory responses with a linear model were computed and performed as described previously [23,30]. Space-time slope is calculated from the linear regression for the temporally parabola-fitted peaks at different locations within the spiking and excitatory RF centers.

DSI was computed as below:

$$DSI=\frac{\;Peak{\;}_{PD}-Peak_{ND}}{\;Peak{\;}_{PD}+Peak_{ND}},$$ (2)

where Peak_PD_ and Peak_ND_ denote the peak responses (firing rates, current, voltage, or simulated response with arbitrary unit) to PD and ND motion, respectively.

To quantify the contributions of individual component flashes to the apparent motion stimulus, multiple linear regression was performed to fit the response to apparent motion using the responses to each flash.

No statistical method was used to predetermine sample size. All data are reported as the mean ± standard error of mean (SEM). Data distribution was in general assumed to be normal and not formally tested; in the figures, data points are shown. Data sets were compared using 1 sample, paired or unpaired *t* test (two-sided). All the statistical details can be found in the figure legends, including the exact meaning and value of *n*. Statistical significance was defined at *p* < 0.05.

## Supporting information

S1 Fig Spatiotemporal profiles of the ON inhibitory responses. **(A)** Schematic diagram of the flash bar stimulus and representative IPSC traces recorded from the same J-RGC in Fig 2A. **(B)** The spatiotemporal profile for the ON inhibitory conductance (G_i_) responses. Similar to Fig 2B. Red, ON response. Data for this figure are in S2 Data.
(PDF)

S2 Fig The characterization of J-RGCs’ excitatory inputs. **(A)** Representative excitatory RFs after bath application of PTX + STR, APV, HEX, and TTX. Scale bar, 200 μm. **(B)** Representative EPSCs during motion stimulus from a J-RGC in JamB-CreER/Ai9/Cx36^f/f^ mice (right) and a J-RGC in the control JamB-CreER/Ai9/Cx36^f/+^ mice (left). Traces are aligned to the estimated time when the leading edge of the moving spot entered the RF center (dotted line). Shaded area around the traces, mean ± SEM, *n* = 16 trials. **(C and D)** Comparison of EPSC amplitudes (**C**) and DSI values (**D**) after removing Cx36 selectively in J-RGCs. Control group includes the data from both Cx36^+/+^ and Cx36^+/−^ mice. Error bars, SEM. In **C**, control group PD vs. ND, paired *t* test, ***, *p* < 0.001, *n* = 24 cells; Cx36^−/−^ group PD vs. ND, paired *t* test, *, *p* < 0.05, *n* = 6 cells; PD from control vs. Cx36^−/−^ group, unpaired *t* test, ***, *p* < 0.001; ND from control vs. Cx36^−/−^ group, unpaired *t* test, **, *p* < 0.01. In **D**, unpaired *t* test; NS, not significant; *n* = 24/6 cells for control/Cx36^−/−^ group. **(E)** Representative EPSCs recorded during motion stimulus before (left) and after (right) bath application of PTX and STR. Shaded area around the traces, mean ± SEM, *n* = 8 trials. **(F and G)** Comparison of EPSC amplitudes (**F**) and total charges (**G**) between PD and ND motion under control and inhibition blocked condition. *n* = 5 cells. Data for this figure are in S2 Data.
(PDF)

S3 Fig Simulation of the Vm change responses during motion using actual or modified synaptic conductance as inputs. **(A)** Simulated J-RGCs’ responses with actual (left) and modified (middle and right) synaptic inputs. Similar to Fig 3B except V_start_ = E_inh_. Shaded area around the traces, mean ± SEM. G_e_, excitatory conductance. G_i_, inhibitory conductance. **(B and C)** Peak depolarization of simulated responses to PD and ND motion under the DS_e_ (**B**) and DS_i_ (**C**) conditions across different V_start_. Responses under the control condition are included for comparison. **(D)** DSI values of simulated responses under control, DS_e_ and DS_i_ conditions across different V_start_. Error bars, SEM. *n* = 100 trials. Data for this figure are in S2 Data.
(PDF)

S4 Fig Representative responses to individual OFF bar flashes at different positions along the PD-ND axis. (PDF)

S5 Fig Apparent motion stimulus evokes similar DS IPSC responses as the smooth motion stimulus. **(A)** Representative OFF IPSC responses evoked by apparent motion stimulus and smooth motion stimulus. **(B)** Comparison of OFF IPSCs evoked by apparent motion stimulus and smooth motion stimulus. Each point on the graph compares the mean IPSC amplitudes of a J-RGC’s response in a 20 ms time bin between smooth motion (horizontal) and apparent motion (vertical). Six J-RGCs’ responses are included. Dashed: line of identity. Data for this figure are in S2 Data.
(PDF)

S6 Fig J-RGCs’ direction selectivity under different drug conditions and luminance levels using the vector sum method. **(A)** Polar plots of a J-RGC’s average spiking responses to motion in 8 directions under control condition and after bath application of PTX and PTX + STR. *n* = 10 trials. **(B)** Polar plots of a J-RGC’s average spiking responses to motion in 8 directions before and after bath application of DCG-IV. *n* = 10 trials. **(C–E)** Comparison of J-RGCs’ direction selectivity measured by the vector sum magnitudes of the motion responses before and after bath application of PTX (**C**, *n* = 6 cells), PTX+STR (**D**, *n* = 8 cells), and DCG-IV (**E**, *n* = 7 cells). Paired *t* test; **, *p* < 0.01; ***, *p* < 0.001. **(F)** Vector sum magnitudes of J-RGCs’ responses to the moving spot stimulus under different luminance levels. Data from 23 randomly chosen OFF RGCs and 23 genetically defined ON-OFF DSGCs [57] are included for reference. *n* = 6/11/3 J-RGCs for optical density = 4.0/3.0/2.0 group. Dotted lines in **C–F**: vector sum magnitude = 0.12, mean + 2 × SD of the OFF RGC group. Error bars, SEM. Data for this figure are in S2 Data.
(PDF)

S7 Fig Hotspots of SAC inputs derived from deconvolution. **(A)** Inputs for the deconvolution. Cyan, distribution of light intensity within the LED spot. Magenta, normalized IPSC peak amplitudes of a J-RGC to the activation of SACs by LED spots centered at different distances from the soma of the J-RGC. **(B)** Hotspots of SAC inputs revealed by deconvolution using the inputs in **A**. **(C)** Comparison of convolved result (convolution of the cyan curve in **A** and connection strength in **B**) and recorded result (the magenta curve in **A**). **(D–F)** The same as **A–C**, except using the data from another example J-RGC for deconvolution. **(G)** A summary for the hotspots of SAC inputs from 6 J-RGCs. Error bars, SEM. **(H)** Comparison of convolved result (convolution of the cyan curve in Fig 6I and connection strength in Fig 6J) and recorded result (the magenta curve in Fig 6I). Data for this figure are in S2 Data.
(PDF)

S8 Fig Direction selectivity of J-RGCs at different speeds. **(A and D)** Representative EPSCs (**A**) and IPSCs (**D**) recorded from a J-RGC during PD and ND motion at 800 μm/s (left) and 2,000 μm/s (right). Traces are aligned to the estimated time when the leading edge of the moving spot entered the RF center (dotted line). Shaded area around the traces, mean ± SEM, *n* = 10 trials. **(B, C, E, and F)** Summary of the peak amplitudes and DSI values of motion evoked OFF EPSCs (**B and C**) and IPSCs (**E and F**). In **B** and **C**, *n* = 13 cells; in **E** and **F**, *n* = 9 cells. **(G)** The spiking responses of a J-RGC to PD and ND motion at 800 μm/s (left) and 2,000 μm/s (right). **(H)** Comparison of DSI values at 800 μm/s and 2,000 μm/s. *n* = 12 cells. Dotted line: DSI = 0.3. **(I)** Polar plots for the average spiking responses of a J-RGC to motion in 8 directions at 800 μm/s and 2,000 μm/s. *n* = 11 trials. **(J)** Comparison of direction selectivity measured by vector sum magnitudes of J-RGCs’ responses to 8 directions of motion at 800 μm/s and 2,000 μm/s. Dotted line: vector sum magnitude = 0.12. *n* = 6 cells. Error bars, SEM. In **B**, **C**, **E**, **F**, **H**, **and J**, paired *t* test; *, *p* < 0.05; **, *p* < 0.01; ***, *p* < 0.001; NS, not significant. Data for this figure are in S2 Data.
(PDF)

S9 Fig I-V relationships of light evoked synaptic inputs to J-RGCs. **(A)** Representative average synaptic currents recorded from a J-RGC under different holding potentials. Traces are the average of 6 repeats, with different colors indicating different holding potentials. Arrow, onset of a dark spot stimulus. **(B)** I-V curve from **A** at the time of peak conductance (magenta dashed line in **A**). This is the I-V relationship for a mixture of all synaptic inputs. **(C and D)** The same as **A** and **B** but with the bath application of PTX + STR to block all the inhibitory inputs. The reversal potential for remaining currents was calculated to be 9.7 ± 3.3 mV, *n* = 3 cells. Data for this figure are in S2 Data.
(PDF)

S10 Fig J-RGCs’ responses to the full-field drifting grating stimulus. **(A)** An example where both the orientation-selective and direction-selective components of the J-RGC’s response could be observed using the drifting grating stimulus. Dashed green line, orientation of the J-RGC dendrites. **(B and C)** A J-RGC’s responses with different degrees of orientation and direction selectivity under 2 drifting grating stimuli: 320 μm period, 640 μm/s in **B**; 160 μm period, 320 μm/s in **C**. The J-RGC dendrite orientations are illustrated in green. Blue arrow, vector sum magnitude.
(PDF)

S11 Fig IPSC responses to the motion stimulus with cholinergic transmission blocked. **(A)** Representative IPSCs recorded during PD (left) and ND (right) motion before (blue) and after (red) bath application of 300 μm HEX to block the cholinergic transmission. Shaded area around the traces, mean ± SEM, n = 16 trials. **(B)** Summary of the effect of HEX on the peak amplitudes of motion evoked IPSCs. Paired *t*-test; *, p < 0.05; NS, not significant; n = 4 cells. Data for this figure are in S2 Data.
(PDF)

S1 Data Underlying data for Figs 1–6. (XLSX)

S2 Data Underlying data for S1–S3 and S5–S9 and S11 Figs. (XLSX)

## Abbreviations

ChR2: channelrhodopsin-2
Cx36: connexin36
DS: direction-selective
DSGC: direction-selective ganglion cell
DSI: direction selectivity index
EPSC: excitatory postsynaptic current
GABA: gamma-aminobutyric acid
HEX: hexamethonium
IPL: inner plexiform layer
IPSC: inhibitory postsynaptic current
J-RGC: J-type retinal ganglion cell
nAChR: nicotinic acetylcholine receptor
ND: null direction
NMDA: N-Methyl-D-aspartic acid
PD: preferred direction
PTX: picrotoxin
RF: receptive field
RGC: retinal ganglion cell
SAC: starburst amacrine cell
STR: strychnine
TTX: tetrodotoxin
