# Supplementary figures and images for: Asymmetric connections with starburst amacrine cells underlie the upward motion selectivity of J-type retinal ganglion cells
Source: PLoS Biol. 2023 Sep 18;21(9):e3002301. doi: 10.1371/journal.pbio.3002301 (PMC10538761; doi:10.1371/journal.pbio.3002301)

**A**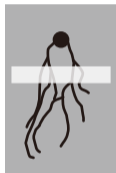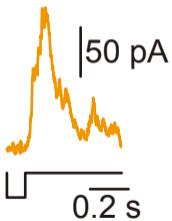**B**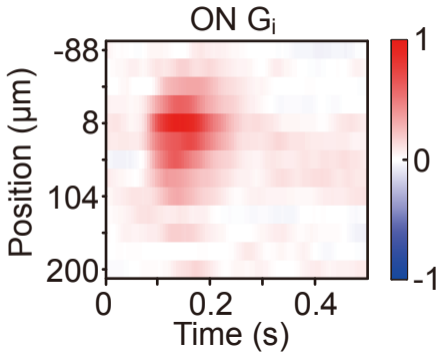

Supplement: S1 Fig — (A) Schematic diagram of the flash bar stimulus and representative IPSC traces recorded from the same J-RGC in Fig 2A. (B) The spatiotemporal profile for the ON inhibitory conductance (Gi) responses. Similar to Fig 2B. Red, ON response. Data for this figure are in S2 Data. (PDF) [file pbio.3002301.s001.pdf]

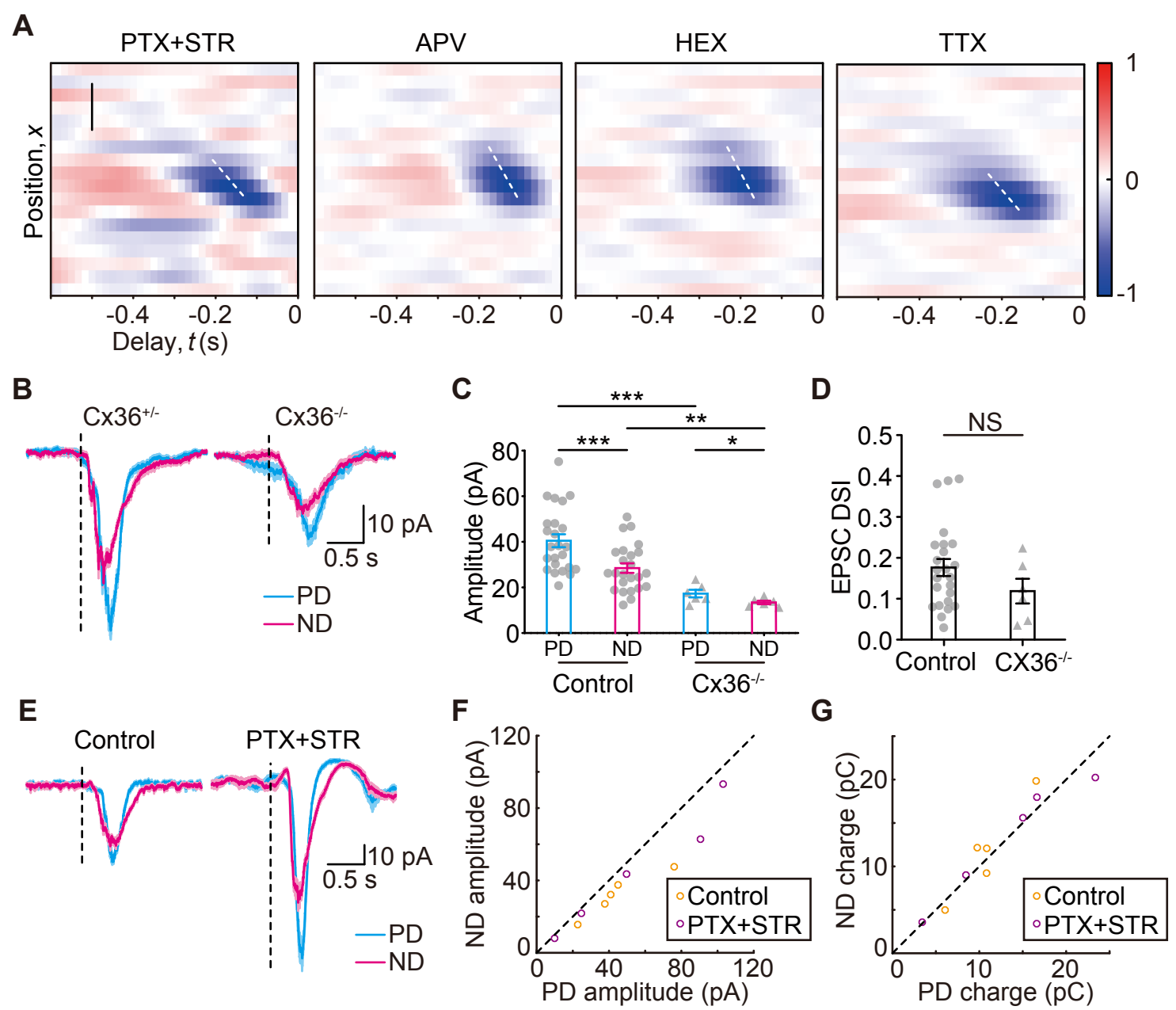

Supplement: S2 Fig — (A) Representative excitatory RFs after bath application of PTX + STR, APV, HEX, and TTX. Scale bar, 200 μm. (B) Representative EPSCs during motion stimulus from a J-RGC in JamB-CreER/Ai9/Cx36f/f mice (right) and a J-RGC in the control JamB-CreER/Ai9/Cx36f/+ mice (left). Traces are aligned to the estimated time when the leading edge of the moving spot entered the RF center (dotted line). Shaded area around the traces, mean ± SEM, n = 16 trials. (C and D) Comparison of EPSC amplitudes (C) and DSI values (D) after removing Cx36 selectively in J-RGCs. Control group includes the data from both Cx36+/+ and Cx36+/− mice. Error bars, SEM. In C, control group PD vs. ND, paired t test, ***, p < 0.001, n = 24 cells; Cx36−/− group PD vs. ND, paired t test, *, p < 0.05, n = 6 cells; PD from control vs. Cx36−/− group, unpaired t test, ***, p < 0.001; ND from control vs. Cx36−/− group, unpaired t test, **, p < 0.01. In D, unpaired t test; NS, not significant; n = 24/6 cells for control/Cx36−/− group. (E) Representative EPSCs recorded during motion stimulus before (left) and after (right) bath application of PTX and STR. Shaded area around the traces, mean ± SEM, n = 8 trials. (F and G) Comparison of EPSC amplitudes (F) and total charges (G) between PD and ND motion under control and inhibition blocked condition. n = 5 cells. Data for this figure are in S2 Data. (PDF) [file pbio.3002301.s002.pdf]

**A**Control (DS  $G_e$ +DS  $G_i$ )DS<sub>e</sub> (DS  $G_e$ +mean  $G_i$ )DS<sub>i</sub> (DS  $G_i$ +mean  $G_e$ )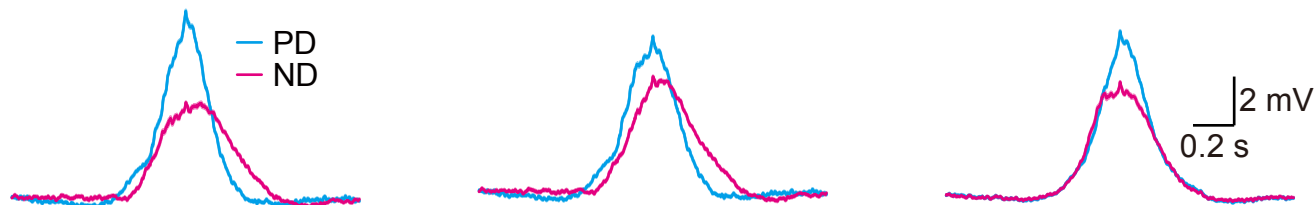**B**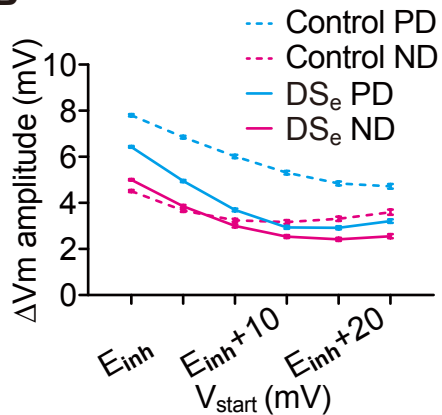**C**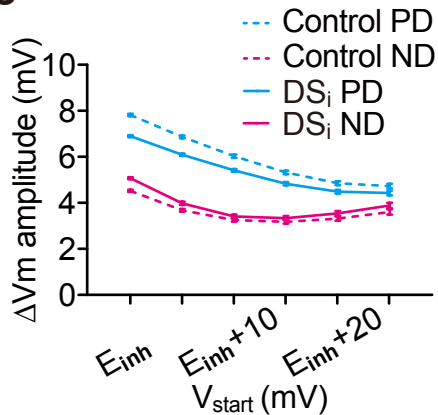**D**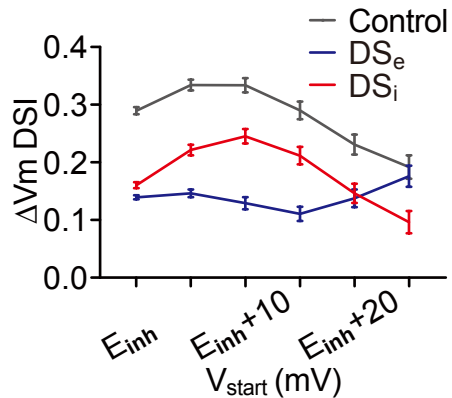

Supplement: S3 Fig — (A) Simulated J-RGCs’ responses with actual (left) and modified (middle and right) synaptic inputs. Similar to Fig 3B except Vstart = Einh. Shaded area around the traces, mean ± SEM. Ge, excitatory conductance. Gi, inhibitory conductance. (B and C) Peak depolarization of simulated responses to PD and ND motion under the DSe (B) and DSi (C) conditions across different Vstart. Responses under the control condition are included for comparison. (D) DSI values of simulated responses under control, DSe and DSi conditions across different Vstart. Error bars, SEM. n = 100 trials. Data for this figure are in S2 Data. (PDF) [file pbio.3002301.s003.pdf]

Bar 1

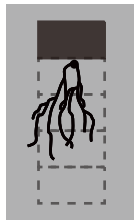

Bar 2  
(Proximal)

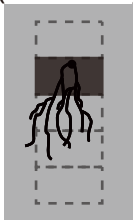

Bar 3  
(Distal)

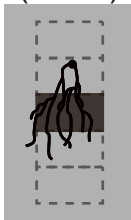

Bar 4

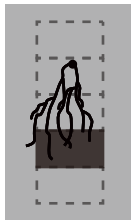

Bar 5

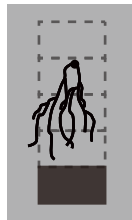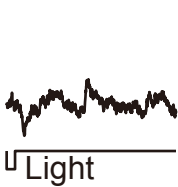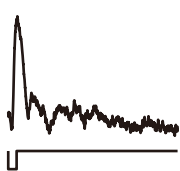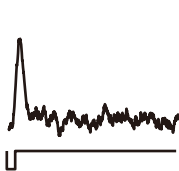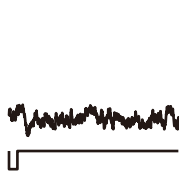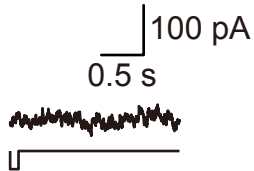

Supplement: S4 Fig — (PDF) [file pbio.3002301.s004.pdf]

**A**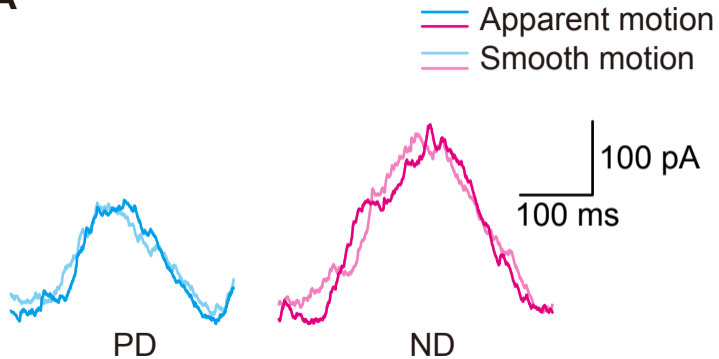**B**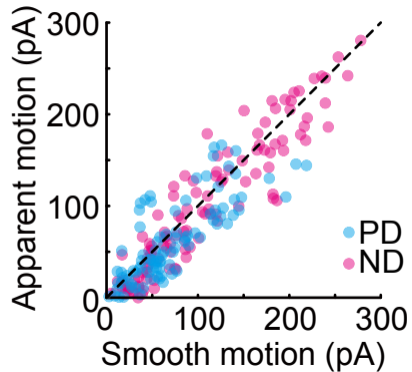

Supplement: S5 Fig — (A) Representative OFF IPSC responses evoked by apparent motion stimulus and smooth motion stimulus. (B) Comparison of OFF IPSCs evoked by apparent motion stimulus and smooth motion stimulus. Each point on the graph compares the mean IPSC amplitudes of a J-RGC’s response in a 20 ms time bin between smooth motion (horizontal) and apparent motion (vertical). Six J-RGCs’ responses are included. Dashed: line of identity. Data for this figure are in S2 Data. (PDF) [file pbio.3002301.s005.pdf]

**A**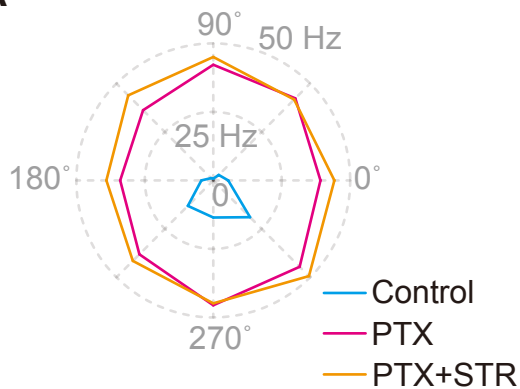**B**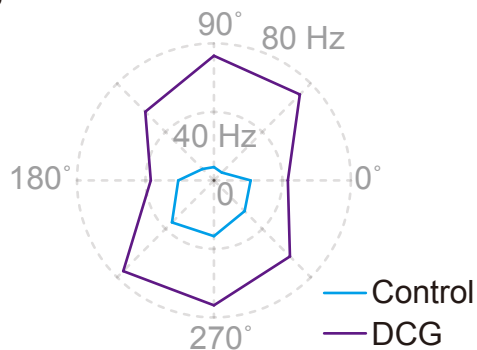**C**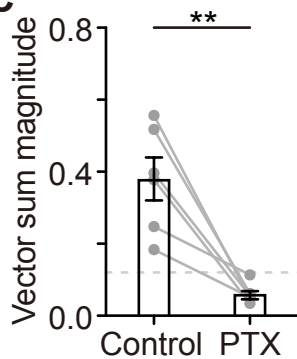**D**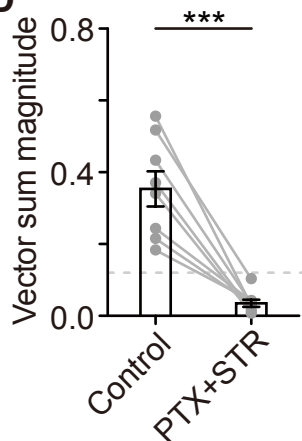**E**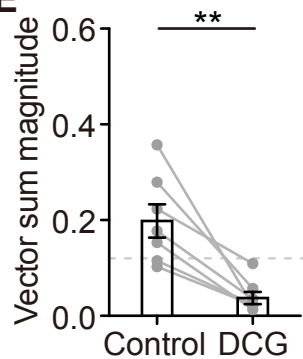**F**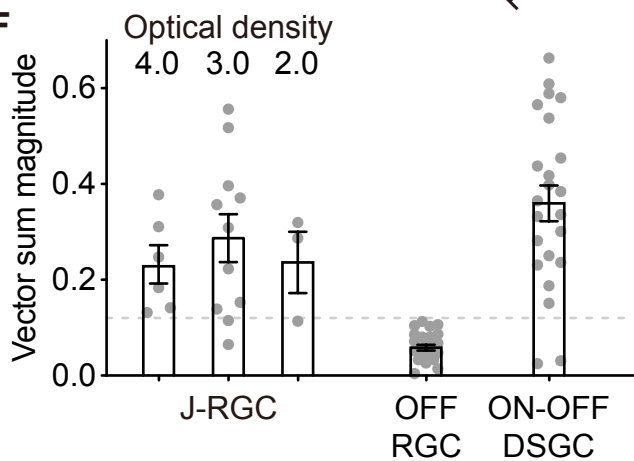

Supplement: S6 Fig — (A) Polar plots of a J-RGC’s average spiking responses to motion in 8 directions under control condition and after bath application of PTX and PTX + STR. n = 10 trials. (B) Polar plots of a J-RGC’s average spiking responses to motion in 8 directions before and after bath application of DCG-IV. n = 10 trials. (C–E) Comparison of J-RGCs’ direction selectivity measured by the vector sum magnitudes of the motion responses before and after bath application of PTX (C, n = 6 cells), PTX+STR (D, n = 8 cells), and DCG-IV (E, n = 7 cells). Paired t test; **, p < 0.01; ***, p < 0.001. (F) Vector sum magnitudes of J-RGCs’ responses to the moving spot stimulus under different luminance levels. Data from 23 randomly chosen OFF RGCs and 23 genetically defined ON-OFF DSGCs [57] are included for reference. n = 6/11/3 J-RGCs for optical density = 4.0/3.0/2.0 group. Dotted lines in C–F: vector sum magnitude = 0.12, mean + 2 × SD of the OFF RGC group. Error bars, SEM. Data for this figure are in S2 Data. (PDF) [file pbio.3002301.s006.pdf]

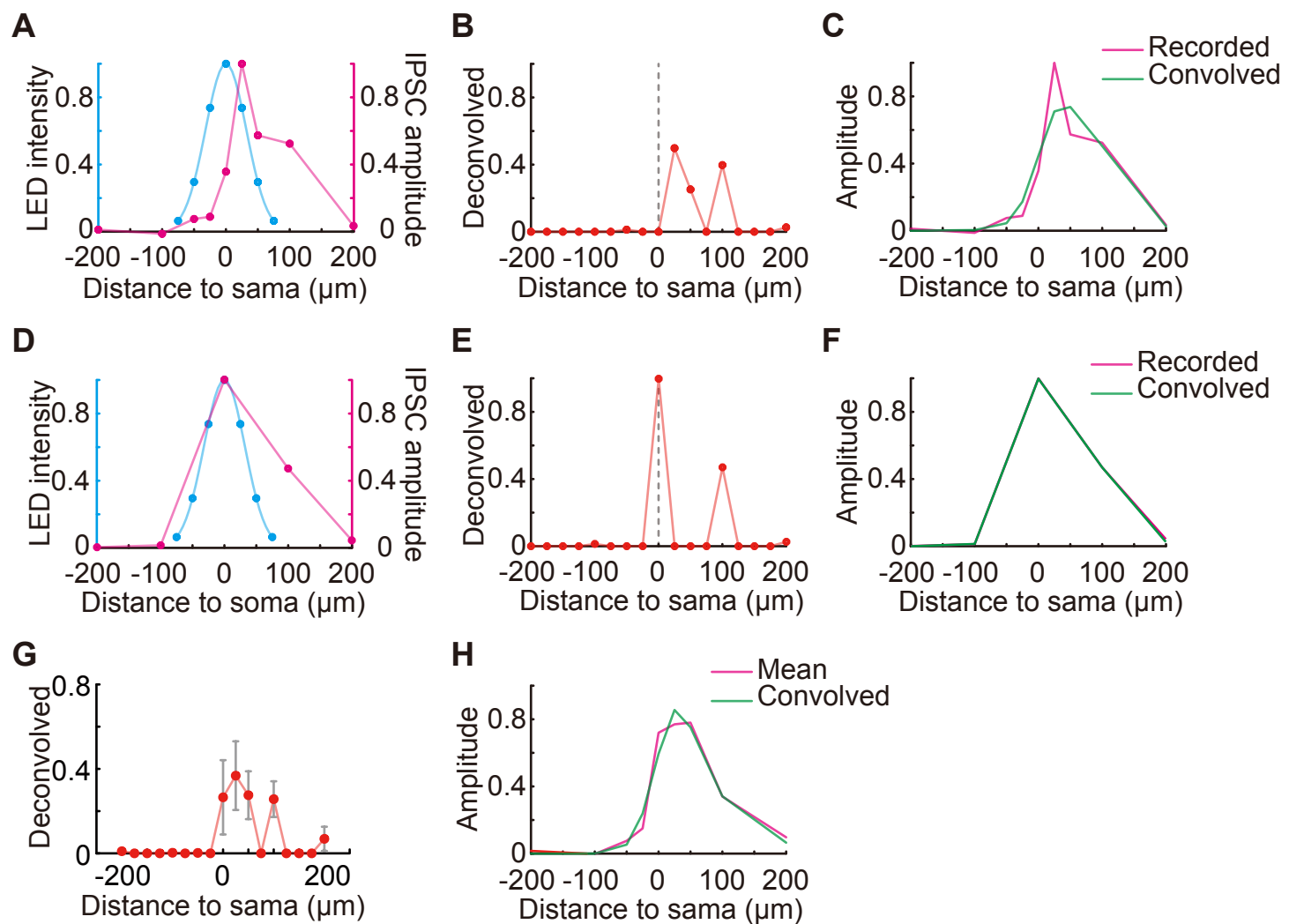

Supplement: S7 Fig — (A) Inputs for the deconvolution. Cyan, distribution of light intensity within the LED spot. Magenta, normalized IPSC peak amplitudes of a J-RGC to the activation of SACs by LED spots centered at different distances from the soma of the J-RGC. (B) Hotspots of SAC inputs revealed by deconvolution using the inputs in A. (C) Comparison of convolved result (convolution of the cyan curve in A and connection strength in B) and recorded result (the magenta curve in A). (D–F) The same as A–C, except using the data from another example J-RGC for deconvolution. (G) A summary for the hotspots of SAC inputs from 6 J-RGCs. Error bars, SEM. (H) Comparison of convolved result (convolution of the cyan curve in Fig 6I and connection strength in Fig 6J) and recorded result (the magenta curve in Fig 6I). Data for this figure are in S2 Data. (PDF) [file pbio.3002301.s007.pdf]

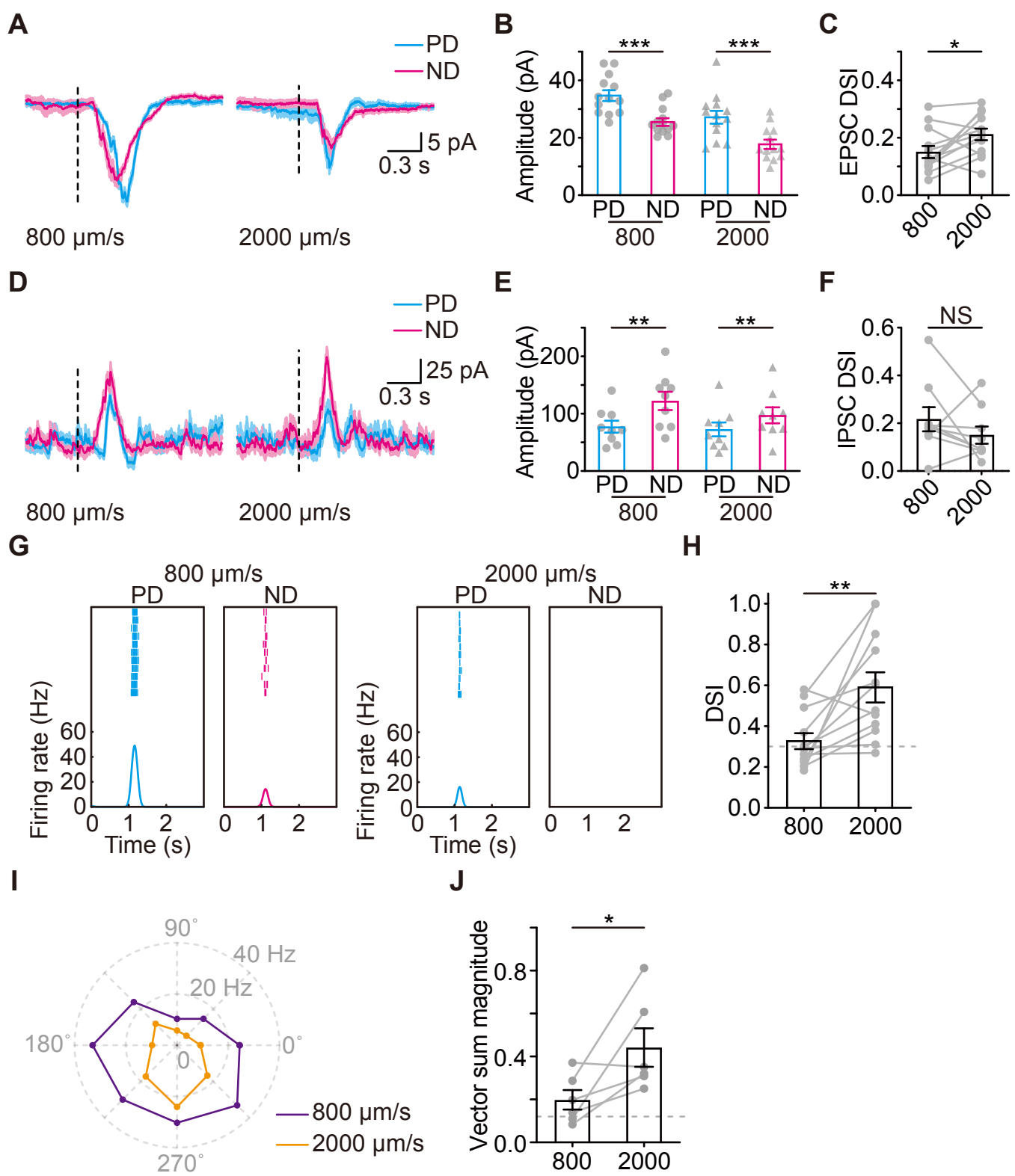

Supplement: S8 Fig — (A and D) Representative EPSCs (A) and IPSCs (D) recorded from a J-RGC during PD and ND motion at 800 μm/s (left) and 2,000 μm/s (right). Traces are aligned to the estimated time when the leading edge of the moving spot entered the RF center (dotted line). Shaded area around the traces, mean ± SEM, n = 10 trials. (B, C, E, and F) Summary of the peak amplitudes and DSI values of motion evoked OFF EPSCs (B and C) and IPSCs (E and F). In B and C, n = 13 cells; in E and F, n = 9 cells. (G) The spiking responses of a J-RGC to PD and ND motion at 800 μm/s (left) and 2,000 μm/s (right). (H) Comparison of DSI values at 800 μm/s and 2,000 μm/s. n = 12 cells. Dotted line: DSI = 0.3. (I) Polar plots for the average spiking responses of a J-RGC to motion in 8 directions at 800 μm/s and 2,000 μm/s. n = 11 trials. (J) Comparison of direction selectivity measured by vector sum magnitudes of J-RGCs’ responses to 8 directions of motion at 800 μm/s and 2,000 μm/s. Dotted line: vector sum magnitude = 0.12. n = 6 cells. Error bars, SEM. In B, C, E, F, H, and J, paired t test; *, p < 0.05; **, p < 0.01; ***, p < 0.001; NS, not significant. Data for this figure are in S2 Data. (PDF) [file pbio.3002301.s008.pdf]

**A**

control

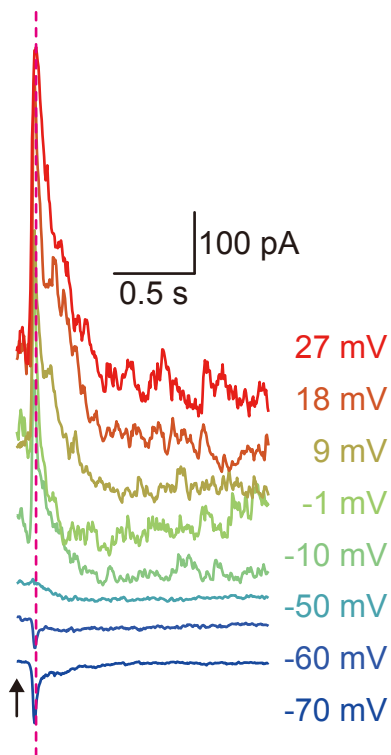**C**

PTX+STR

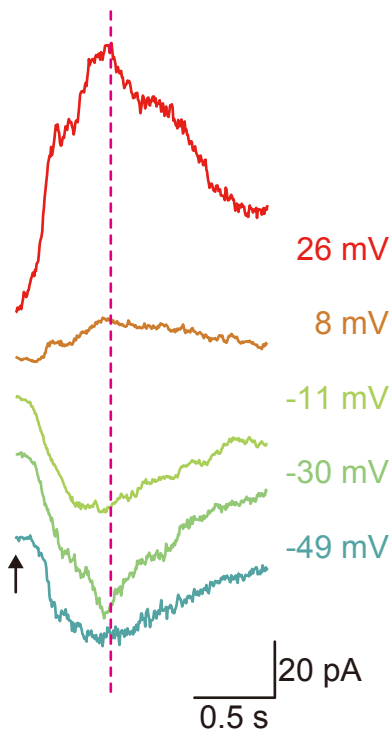**B**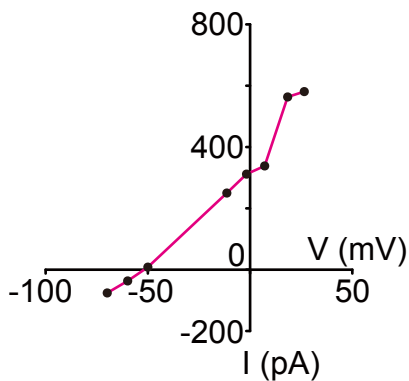**D**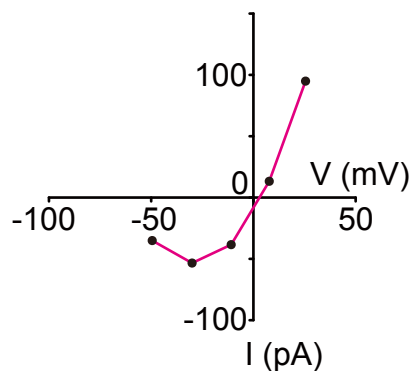

Supplement: S9 Fig — (A) Representative average synaptic currents recorded from a J-RGC under different holding potentials. Traces are the average of 6 repeats, with different colors indicating different holding potentials. Arrow, onset of a dark spot stimulus. (B) I-V curve from A at the time of peak conductance (magenta dashed line in A). This is the I-V relationship for a mixture of all synaptic inputs. (C and D) The same as A and B but with the bath application of PTX + STR to block all the inhibitory inputs. The reversal potential for remaining currents was calculated to be 9.7 ± 3.3 mV, n = 3 cells. Data for this figure are in S2 Data. (PDF) [file pbio.3002301.s009.pdf]

**A**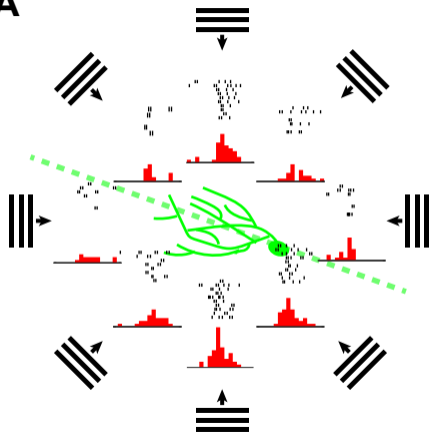**B**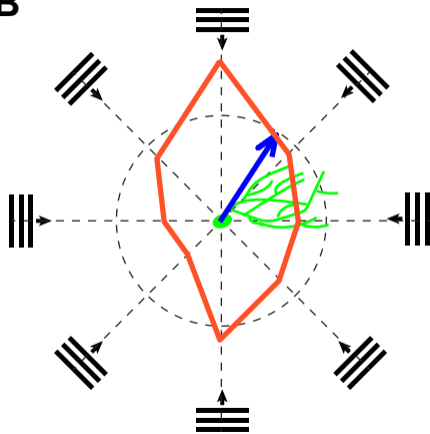**C**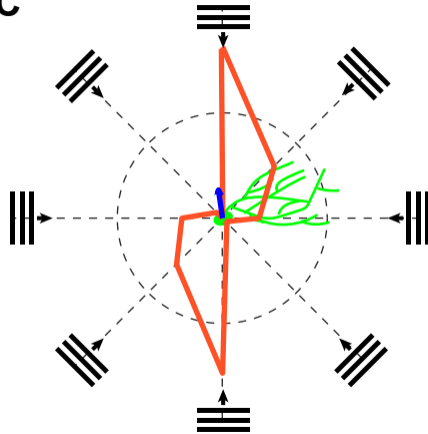

Supplement: S10 Fig — (A) An example where both the orientation-selective and direction-selective components of the J-RGC’s response could be observed using the drifting grating stimulus. Dashed green line, orientation of the J-RGC dendrites. (B and C) A J-RGC’s responses with different degrees of orientation and direction selectivity under 2 drifting grating stimuli: 320 μm period, 640 μm/s in B; 160 μm period, 320 μm/s in C. The J-RGC dendrite orientations are illustrated in green. Blue arrow, vector sum magnitude. (PDF) [file pbio.3002301.s010.pdf]

**A**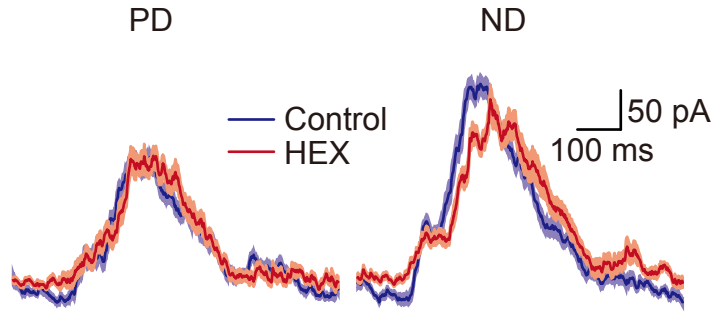**B**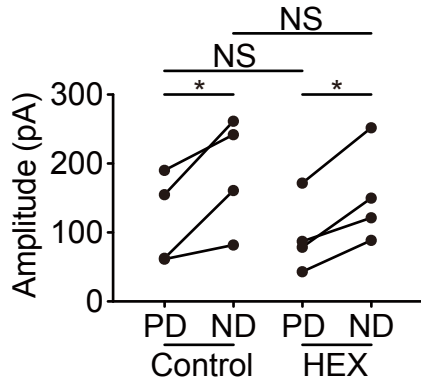

Supplement: S11 Fig — (A) Representative IPSCs recorded during PD (left) and ND (right) motion before (blue) and after (red) bath application of 300 μm HEX to block the cholinergic transmission. Shaded area around the traces, mean ± SEM, n = 16 trials. (B) Summary of the effect of HEX on the peak amplitudes of motion evoked IPSCs. Paired t-test; *, p < 0.05; NS, not significant; n = 4 cells. Data for this figure are in S2 Data. (PDF) [file pbio.3002301.s011.pdf]
